# Serial amplitude-integrated electroencephalography trajectories and brain-injury-associated functional immaturity in extremely preterm infants

**DOI:** 10.3389/fped.2026.1912111

**Published:** 2026-08-26

**Authors:** Yong Zhang, Dekai Xu, Guoyong Luo, Yongping Xu, Yujing Yang, Yong Ji

**Affiliations:** Neonatal Intensive Care Unit, Children’s Hospital of Shanxi Province, Taiyuan, Shanxi, China

**Keywords:** amplitude-integrated electroencephalography, brain injury, Burdjalov maturation score, extremely preterm infants, intraventricular hemorrhage, longitudinal cohort, postmenstrual age, white-matter injury

## Abstract

**Objective:**

To characterize postmenstrual-age (PMA)-specific Burdjalov maturation trajectories in extremely preterm infants and to evaluate how imaging-defined major brain injury was associated with these trajectories and with apparent within-cohort discrimination between imaging-defined groups across PMA windows.

**Methods:**

We conducted a single-center retrospective cohort study of 156 infants born before 28 weeks’ gestation, comprising 109 infants without major brain injury (reference group) and 47 with brain injury, defined as grade III–IV intraventricular hemorrhage or white-matter injury. These infants contributed 723 analyzable serial amplitude-integrated electroencephalography (aEEG) recordings across PMA 26 to 36 weeks. Burdjalov scores were analyzed using linear mixed-effects models, categorical-PMA contrasts, a patient-clustered ordinal model, complete time-window sensitivity analyses, and joint patient-level bootstrap comparisons of PMA-specific areas under the curve.

**Results:**

The Burdjalov total score increased by 0.92 points per week of PMA. Infants with brain injury scored on average 1.45 points lower at PMA 32 weeks (95% confidence interval −1.65 to −1.25). Although the continuous-scale model produced a positive PMA-by-group interaction, its magnitude and statistical significance varied across analysis windows and it was not reproduced by the ordinal model (*p* = 0.979). Categorical-PMA estimates showed a non-monotonic pattern across the full observation period: the imprecise difference at PMA 26 weeks was followed by the largest observed difference at PMA 28 weeks and progressively smaller differences thereafter. At PMA 36 weeks, 87.4% of reference-group and 27.3% of brain-injury recordings reached the maximum score. The largest observed area under the curve was at PMA 32 weeks (0.886, 95% confidence interval 0.824 to 0.940), but joint patient-level bootstrap comparisons did not distinguish PMA 32 from PMA 28, 30, or 36 weeks.

**Conclusions:**

Imaging-defined major brain injury was associated with persistently lower Burdjalov scores. The unstable linear interaction on this bounded scale should not be interpreted as biological recovery or catch-up maturation. The retrospective within-cohort discrimination estimates do not establish predictive, diagnostic, screening, or clinical decision utility.

## 1 Introduction

Extremely preterm infants, those born before 28 completed weeks of gestation, undergo a period of intense cortical organization, white-matter development, and emerging cerebral state regulation while exposed to substantial clinical and biological stress. Major brain injury in this population, including severe intraventricular hemorrhage and white-matter injury, is closely linked to impaired brain maturation and to adverse neurodevelopmental outcomes across childhood (1, 2). Cranial ultrasound and structural magnetic resonance imaging are indispensable for defining structural injury, but they do not directly capture the dynamic process of functional cerebral maturation across the neonatal intensive care unit (NICU) course. Bedside tools that serially track functional brain maturation may therefore complement structural imaging in this high-risk population.

Amplitude-integrated electroencephalography (aEEG) is a widely used bedside neuromonitoring modality that provides a simplified, continuous representation of background cerebral activity accessible to non-specialist interpretation (3). The Burdjalov maturation score integrates four ordinal subscores, background continuity, sleep–wake cycling, lower-border amplitude, and bandwidth, into a composite ranging from 0 to 13, with higher values indicating more mature cortical function (4). Both the components and the total increase with advancing postmenstrual age (PMA) and approach near-maximal values around 35 to 36 weeks (4–6). Because functional cortical maturation over this interval is rapid and nonlinear, with resting-state networks and cerebral state organization emerging steeply across the preterm period (7), a single aEEG value is difficult to interpret in isolation. A serial trajectory instead captures the rate and pattern of maturation, allowing an individual score to be read against an expected developmental slope rather than as an isolated abnormality. Consistent with this clinical relevance, single-timepoint aEEG and low early Burdjalov scores have been associated with imaging-defined injury and adverse neurodevelopmental outcomes (8–10), and aEEG assessed near 32 weeks PMA or at term-equivalent age correlates with structural MRI findings and later outcomes (11–14).

Despite this evidence, how Burdjalov-based maturation evolves across successive PMA windows in infants born below 28 weeks, and how imaging-defined major brain injury modifies that trajectory, remain poorly characterized. Most prior work has examined aEEG at single timepoints rather than as a serial trajectory across the NICU course, even though serial aEEG recording is feasible and safe in this population (15). In particular, it is unclear which PMA window provides the greatest separation between injured and reference infants, whether any maturational delay is distributed uniformly across subdomains or concentrated in features such as sleep–wake cycling and bandwidth, and whether the between-group difference persists or attenuates as the reference group approaches the scoring ceiling. To address these gaps, we conducted a single-center retrospective longitudinal cohort study of 156 extremely preterm infants contributing serial aEEG recordings grouped into PMA 26, 28, 30, 32, 34, and 36-week windows, with three aims: (1) to characterize PMA-specific Burdjalov total and subscore trajectories in infants without major brain injury; (2) to determine whether and how imaging-defined major brain injury was associated with deviation from these trajectories, both overall and within each PMA window; and (3) to describe PMA-specific apparent within-cohort discrimination between the two imaging-defined groups.

## 2 Materials and methods

### 2.1 Study design and setting

We conducted a single-center retrospective longitudinal cohort study at the Neonatal Intensive Care Unit of Children's Hospital of Shanxi Province, Taiyuan, Shanxi, China. The study period covered consecutive admissions from November 2020 to October 2025. The protocol was approved by the Medical Ethics Committee of Children's Hospital of Shanxi Province (approval number IRB-WZ-2025-039), and the study was conducted in accordance with the Declaration of Helsinki (16). Because the analysis used de-identified clinical records collected as part of routine bedside neuromonitoring, the requirement for written informed consent was waived. Reporting follows the STROBE recommendations for observational cohort studies (17). In a previous report from our group, Luo et al. (18) described 488 aEEG recordings from 101 infants without major brain injury; these observations overlap with the present cohort. The present study additionally includes 8 reference infants contributing 30 recordings and a distinct brain-injury cohort of 47 infants contributing 205 recordings, and addresses longitudinal differences between imaging-defined groups rather than a single-arm descriptive profile.

### 2.2 Participants

All consecutive extremely preterm infants admitted to the NICU during the study period were screened for eligibility. Inclusion criteria were: (i) gestational age at birth less than 28 completed weeks; and (ii) the availability of at least three bedside amplitude-integrated electroencephalography (aEEG) recordings at postmenstrual age (PMA) 26, 28, 30, 32, 34 or 36 weeks, each lasting at least six hours, a requirement intended to allow an individual longitudinal maturation trajectory to be defined. Exclusion criteria were: (i) major congenital malformation, known chromosomal disorder, or inborn metabolic disease; (ii) hydrocephalus, or meningitis/ventriculitis temporally overlapping any PMA window from which an aEEG recording was included; (iii) necrotizing enterocolitis or severe systemic infection temporally overlapping an included aEEG recording; (iv) exposure to sedative or anesthetic agents within 48 hours before any included aEEG recording; (v) clinical or electrographic seizures; and (vi) incomplete clinical or imaging records precluding outcome ascertainment. Intracranial infection or necrotizing enterocolitis recorded outside all windows contributing an included aEEG recording was not exclusionary; the temporal relationship was verified during data abstraction. These criteria were applied to isolate the maturational aEEG signal rather than to maximize cohort representativeness, and the consequent limitations are addressed below.

**Figure 1:**
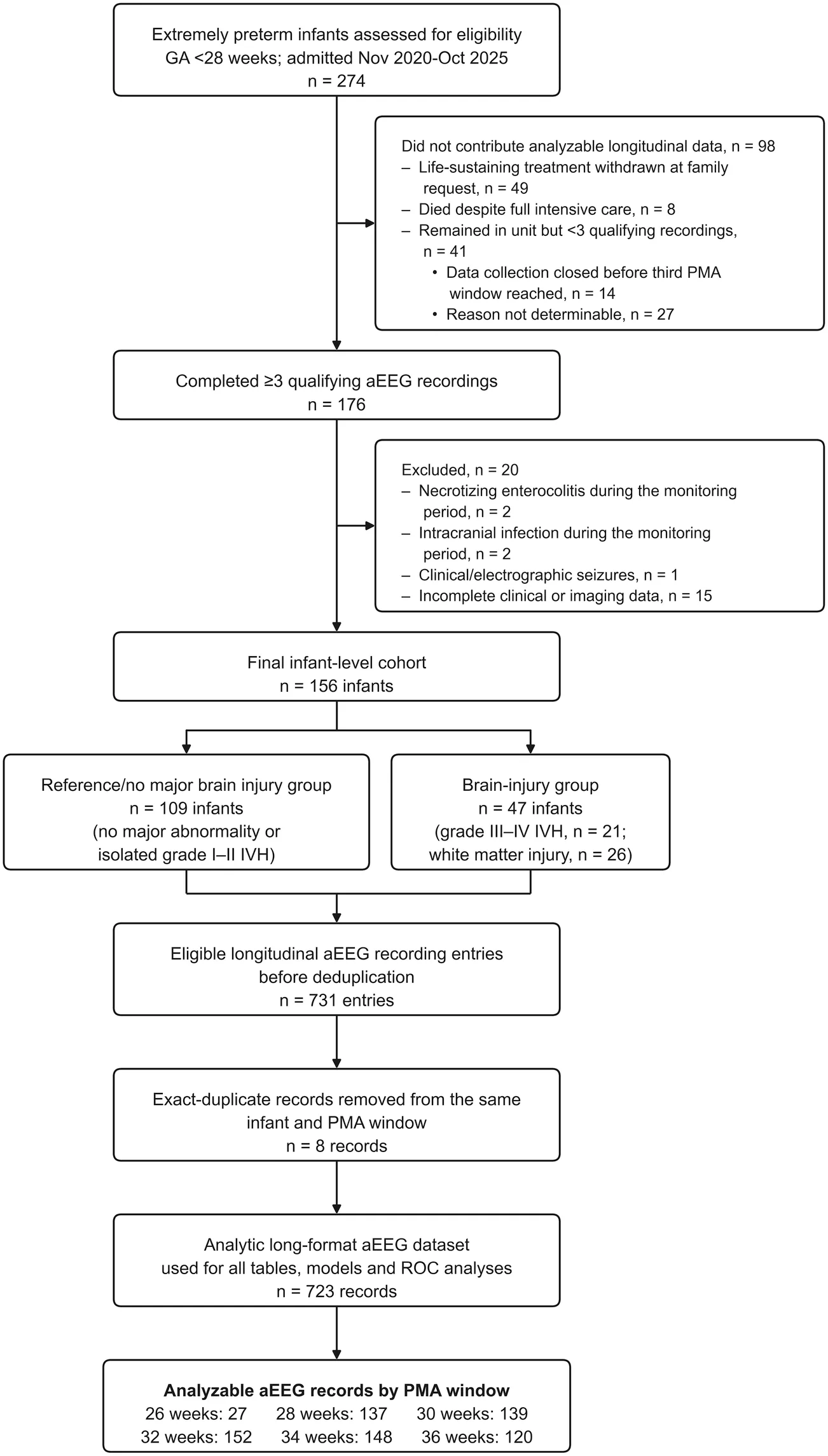
Study flow and longitudinal aEEG data availability. Of 274 infants born before 28 completed weeks of gestation and admitted between November 2020 and October 2025, 98 did not contribute analyzable longitudinal data: life-sustaining treatment was withdrawn at the family's request in 49, 8 died despite full intensive care, and 41 remained in the unit without accumulating three qualifying recordings. In 14 of these 41 infants, data collection closed before a third postmenstrual-age window was reached; in the remaining 27, the reason could not be reliably reconstructed from the retrospective record. No infant was transferred to another facility or discharged before completion of care. The remaining 176 infants underwent formal eligibility assessment; 20 were excluded (necrotizing enterocolitis during the monitoring period, *n* = 2; intracranial infection during the monitoring period, *n* = 2; clinical or electrographic seizures, *n* = 1; incomplete clinical or imaging data, *n* = 15), leaving 156 infants (109 reference/no major brain injury and 47 brain injury, including 21 with grade III–IV intraventricular hemorrhage and 26 with white-matter injury). These infants contributed 731 eligible recording entries before removal of 8 exact duplicates from the same infant and PMA window, leaving 723 analyzable records. The final per-PMA distribution was 27, 137, 139, 152, 148, and 120 recordings at PMA 26, 28, 30, 32, 34, and 36 weeks, respectively. aEEG, amplitude-integrated electroencephalography; IVH, intraventricular hemorrhage; PMA, postmenstrual age; WMI, white-matter injury.

Of 274 infants born before 28 completed weeks of gestation and admitted during the study period, 98 (35.8%) did not contribute analyzable longitudinal data. Life-sustaining treatment was withdrawn at the family's request in 49 infants (17.9%), 8 infants (2.9%) died despite full intensive care, and 41 remained in the unit but did not accumulate at least three qualifying recordings. Among these 41 infants, data collection closed before the third PMA window was reached in 14; in the remaining 27, the reason could not be reliably reconstructed from the retrospective record. No infant was transferred to another facility or discharged before completion of care. The remaining 176 infants with at least three qualifying recordings underwent formal eligibility assessment. After excluding 20 infants (2 with necrotizing enterocolitis during the monitoring period, 2 with intracranial infection during the monitoring period, 1 with clinical or electrographic seizures, and 15 with incomplete clinical or imaging data), 156 infants were included in the final analytic cohort. Eligible infants were classified into a brain-injury group with grade III–IV intraventricular hemorrhage or white-matter injury and a reference/no major brain injury group without major neuroimaging abnormality. The final cohort comprised 109 reference infants and 47 brain-injury infants. These infants contributed 731 eligible longitudinal aEEG recording entries before deduplication. After removal of 8 exact-duplicate records from the same infant and PMA window, 723 analyzable records remained (Figure 1; Supplementary Tables S2 and S19; and Supplementary Figure S3).

### 2.3 aEEG acquisition

Bedside aEEG was recorded using the Nicolet monitor system (Natus Medical Incorporated, Pleasanton, CA, USA). Nine electrodes (Fp1, Fp2, C3, C4, Cz, T3, T4, O1 and O2) were placed according to the international 10–20 system (3). Electrode impedance was maintained below 20 k*Ω*. Signals were processed using a 0.5–70 Hz band-pass filter, and 50-Hz power-line interference was removed. The aEEG trace was displayed in a semi-logarithmic format at 6 cm/h. Each recording lasted at least six hours. Although the full nine-electrode neonatal montage described above was applied during acquisition, Burdjalov scoring was performed on the single bipolar C3–C4 derivation, as in the original scoring system (4). During the study period, serial aEEG monitoring formed part of the NICU's routine neuromonitoring practice for infants born before 28 completed weeks of gestation. Recordings were scheduled at PMA 26, 28, 30, 32, 34, and 36 weeks during the NICU stay, when monitoring was clinically feasible. Eligibility of a recording for the present analysis was determined by the prespecified PMA window, minimum recording duration, and predefined study criteria, rather than by suspected seizures or acute neurological deterioration. Because recordings could only be obtained while infants remained hospitalized and monitoring was clinically feasible, not every infant contributed data at every PMA window. The observation schedule ended at PMA 36 weeks because monitoring was embedded in inpatient NICU care. Most surviving infants were discharged after this time, and a standardized outpatient aEEG follow-up program was not available during the study period. In addition, the Burdjalov total score approached its upper bound by PMA 35–36 weeks, limiting its resolution near term-equivalent age. Recordings were of sufficient duration to assess background continuity, lower-border amplitude, bandwidth, and sleep–wake cycling when present. Obvious artifactual segments arising from movement, electrode-impedance fluctuation, or caregiving procedures were excluded from visual interpretation before scoring.

### 2.4 Burdjalov maturation scoring

Each aEEG recording was scored according to the Burdjalov maturation scale (4), which integrates four ordinal subscores reflecting distinct domains of functional brain maturation: continuity of the background pattern (range 0–2), sleep–wake cycling (range 0–5), amplitude of the lower border of the trace (range 0–2), and bandwidth of the upper and lower margins (range 0–4). The four subscores are summed to a composite total ranging from 0 to 13, with higher values indicating more mature cortical function. The original 723 recordings were scored retrospectively in one centralized study-phase exercise by a clinician experienced in neonatal aEEG interpretation; scores were not accrued serially across the 2020–2025 enrollment period. aEEG scoring and neuroimaging-based group classification were performed independently. Tracings were de-identified, and the assessor had no access to infant identifiers, clinical information, cranial ultrasound or magnetic resonance imaging findings, or final brain-injury group allocation. The Burdjalov total and subdomain scores were recorded before linkage with the clinical and neuroimaging dataset.

To ensure internal consistency, the analytic total score was computed as the algebraic sum of the four subscores; exact-duplicate record handling is described in section 2.2 and Figure 1.

For the reliability substudy, a second assessor independently re-scored the same 219 recordings from 45 infants (33 reference and 12 brain-injury infants) spanning all six PMA windows. The second assessor was masked to imaging findings, group allocation, and archived original scores. Inter-rater reliability compared the archived study scores with the second assessor's independent scores. Formal intra-rater repeatability was not assessed.

### 2.5 Outcome ascertainment (brain injury)

Brain injury was defined *a priori* from serial neuroimaging obtained during the NICU admission. The first cranial ultrasound was performed within 72 hours of admission, with follow-up ultrasound examinations repeated during the admission at intervals determined by the clinical course rather than a fixed schedule, and magnetic resonance imaging was obtained at approximately 36 weeks' corrected gestational age or before discharge when clinically feasible. Because white-matter injury and periventricular leukomalacia may not be apparent on early cranial ultrasound and typically evolve over the first postnatal weeks, serial cranial ultrasound through the admission and magnetic resonance imaging near 36 weeks were used to capture evolving and established injury. Infants with grade III–IV intraventricular hemorrhage by the Papile classification (19), or with radiologically reported white-matter injury (including periventricular leukomalacia when reported) (20), were classified into the brain-injury group; this group comprised 21 infants with grade III–IV intraventricular hemorrhage (grade III, *n* = 15; grade IV, *n* = 6) and 26 infants with white-matter injury. The reference group comprised 9 infants with normal imaging, 3 with isolated grade I intraventricular hemorrhage, and 97 with isolated grade II intraventricular hemorrhage, without parenchymal involvement or white-matter injury (Supplementary Table S18). Neuroimaging findings were abstracted from archived clinical radiology reports, and group classification was completed before statistical analysis of aEEG scores.

### 2.6 Covariates

Baseline perinatal characteristics were abstracted from the electronic medical record: gestational age at birth, birth weight, infant sex, mode of delivery, antenatal corticosteroid exposure, and conception by assisted reproductive technology. Postnatal systemic sepsis diagnoses and maternal infection/chorioamnionitis were additionally abstracted for descriptive analysis. Complete timing relative to each aEEG recording and serial inflammatory-biomarker trajectories were not uniformly available, and postnatal infection could lie on the pathway between clinical illness and brain injury; these inflammatory exposures were therefore not added *post hoc* to the primary longitudinal model. Other postnatal events occurring after the first eligible aEEG recording were likewise not used as covariates to avoid mediator-adjustment bias (21).

Small-for-gestational-age (SGA) status was defined as birth weight below the sex- and gestational-age-specific 10th percentile. The primary classification used the Chinese WS/T 800–2022 standard (22), which applies from 24 to 42 completed weeks; one infant born at 23 + 6 weeks was therefore unclassifiable by this standard. A secondary classification using Fenton 2025 (23), applicable from 22 to 42 weeks, was used for sensitivity analysis and classified all 156 infants. SGA was not included in the primary model.

### 2.7 Statistical analysis

#### 2.7.1 Descriptive statistics

**Figure 2:**
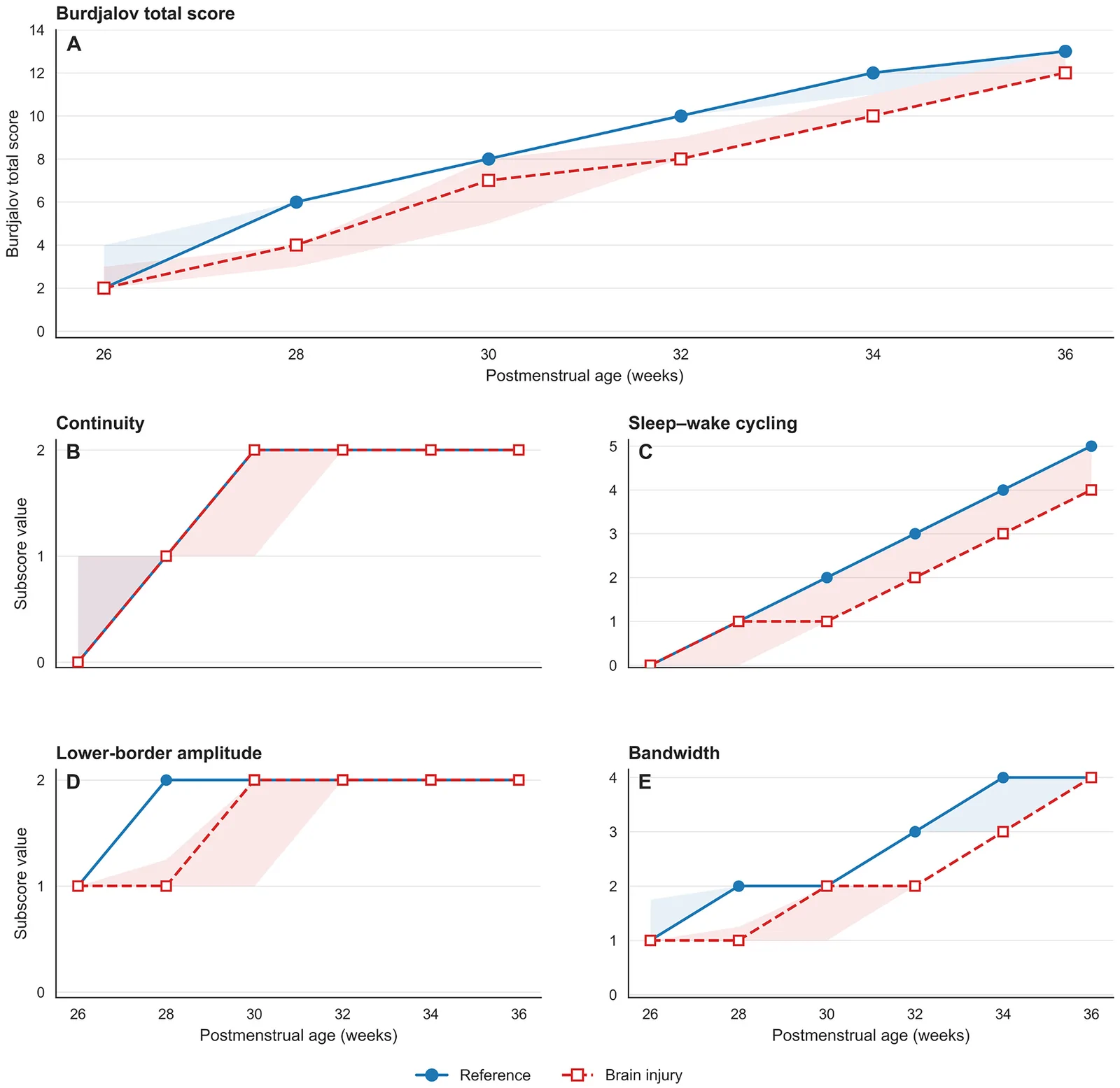
Burdjalov total and subdomain maturation trajectories by group across PMA 26–36 weeks. Group-specific median trajectories and interquartile ranges are shown for the Burdjalov total score and four subdomains. Panel **A** shows the Burdjalov total score (range 0–13). Panels **B–E** show continuity (range 0–2), sleep–wake cycling (range 0–5), lower-border amplitude (range 0–2), and bandwidth (range 0–4), respectively. The reference/no major brain injury group showed progressive maturation toward ceiling values, whereas the brain-injury group followed a similarly increasing but consistently lower trajectory. Continuity and lower-border amplitude reached ceiling values earlier, whereas sleep–wake cycling and bandwidth showed more persistent between-group separation. Descriptive total-score values are provided in Supplementary Table S7, subdomain values in Supplementary Table S3, and per-PMA p-values in Supplementary Table S1. IQR, interquartile range; PMA, postmenstrual age.

Given the bounded and ordinal nature of the aEEG total and subdomain scores, continuous variables were summarized as median (interquartile range, IQR) and categorical variables as count (percentage) throughout. Group differences in baseline characteristics were tested using the Mann–Whitney *U*-test for continuous variables and the *χ*^2^ or Fisher exact test for categorical variables, as appropriate (Table 1). Burdjalov total and subscore values at each PMA window are presented as group-specific medians (IQR), with between-group differences at each PMA tested using the Mann–Whitney *U*-test (Supplementary Tables S7 and S3; trajectories shown in Figure 2).

**Table 1:** Baseline perinatal characteristics by group.

Variable	Reference (*n* = 109)	Brain injury (*n* = 47)	P-value
Gestational age, weeks	26.0 (26.0, 27.0)	26.0 (26.0, 27.0)	0.727
Birth weight, g	900 (800, 1000)	900 (780, 1010)	0.889
Male sex, n (%)	58 (53.2)	20 (42.6)	0.295
Antenatal corticosteroids, n (%)	66 (60.6)	27 (57.4)	0.853
Cesarean delivery, n (%)	44 (40.4)	16 (34.0)	0.572
Assisted reproduction (IVF), n (%)	26 (23.9)	12 (25.5)	0.983
Small for gestational age (WS/T 800–2022), n (%)	5 (4.6)†	5 (10.6)	0.296

Values are median (interquartile range) for continuous variables and n (%) for categorical variables. P-values from Mann–Whitney *U*-test for continuous variables and *χ*^2^ or Fisher exact test for categorical variables. IVF, *in vitro* fertilization. † Denominator=108 for the reference group: one infant born at 23 + 6 weeks fell outside the lower gestational-age limit of the WS/T 800–2022 standard and was therefore not classifiable by this variable.

#### 2.7.2 Primary inferential model

The longitudinal evolution of the Burdjalov total score was modeled using a linear mixed-effects model fitted by restricted maximum likelihood, with PMA centered at 32 weeks, brain-injury group, and the PMA×group interaction as fixed effects and a patient-specific random intercept. A random-intercept-plus-random-slope model was fitted as a covariance-structure diagnostic; it converged but had a near-singular covariance matrix with an intercept–slope correlation of −0.995 and was not used for inference. Conditional residual-versus-fitted and normal Q–Q plots were examined overall and within each PMA window, supplemented by descriptive Brown–Forsythe tests of residual dispersion (Supplementary Figure S6 and Table S16). To demonstrate the dependence of variance partitioning on the fixed-effect specification, random-intercept variance ratios were calculated for intercept-only, PMA-only, PMA-plus-group, and full interaction models (Supplementary Table S8); these ratios were not interpreted as scoring reliability.

#### 2.7.3 Robustness checks

**Figure 3:**
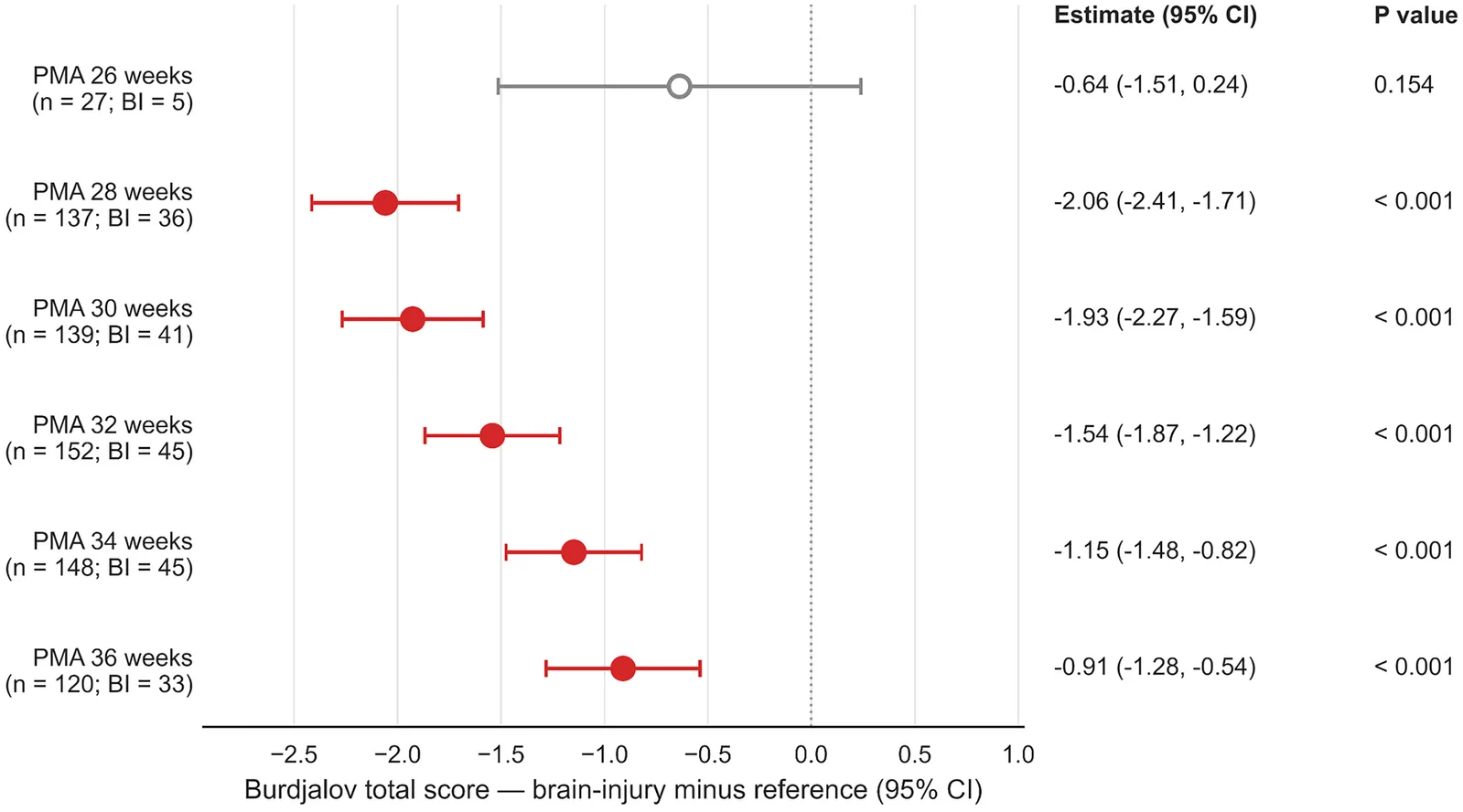
PMA-specific between-group differences in Burdjalov total score. Forest plot of model-estimated group differences from the categorical-PMA linear mixed-effects interaction model. Each estimate represents the brain-injury group minus the reference group at the indicated PMA window; negative values indicate lower scores in the brain-injury group. Horizontal bars indicate 95% confidence intervals; the dashed vertical line denotes the null effect. Filled red markers indicate contrasts with 95% confidence intervals excluding zero; the open grey marker (PMA 26 weeks) indicates a non-significant contrast. Numerical values are reported in Supplementary Table S6. CI, confidence interval; PMA, postmenstrual age.

A Gaussian generalized estimating equation with exchangeable working correlation and robust sandwich standard errors was fitted as a non-random-effects sensitivity analysis (24). Because the total score is bounded and ordinal, a cumulative ordinal GEE with working-independence correlation and patient-clustered robust standard errors was also fitted. The PMA×group estimate was examined across all relevant windows (PMA 26–36, 26–34, 28–34, 26–32, 28–32, and 30–32 weeks) without selecting a window according to statistical significance. Per-PMA between-group differences were obtained from a categorical-PMA interaction model, which avoided imposing a single linear interaction across a potentially non-monotonic trajectory (Figure 3; Supplementary Tables S6 and S13). Exact and near-ceiling frequencies were summarized by PMA and imaging group (Supplementary Table S14 and Figure S7).

#### 2.7.4 Subscore models

Each of the four Burdjalov subscores (continuity, sleep–wake cycling, lower border, bandwidth) was modeled separately using a parallel LMM specification (Supplementary Table S4). Because the four subscores are bounded ordinal scales and several reach ceiling within the observation window, subscore models are interpreted descriptively, alongside the per-PMA Mann–Whitney comparisons reported in Supplementary Table S1.

#### 2.7.5 Exploratory injury-subtype analysis

To assess potential neurophysiological heterogeneity within the composite brain-injury group, an exploratory subgroup analysis comparing infants with severe (grade III–IV) intraventricular hemorrhage (*n* = 21) and infants with white-matter injury (*n* = 26) was performed. Group-specific Burdjalov total-score trajectories were compared across PMA windows using the Kruskal–Wallis test across the three groups (reference, severe intraventricular hemorrhage, and white-matter injury) and the Mann–Whitney *U*-test for the severe intraventricular hemorrhage versus white-matter injury contrast. Because of the modest subgroup sizes, this analysis was pre-specified as exploratory and is reported in the Supplementary Tables S10–S12 and Figure S5.

#### 2.7.6 Discrimination

**Figure 4:**
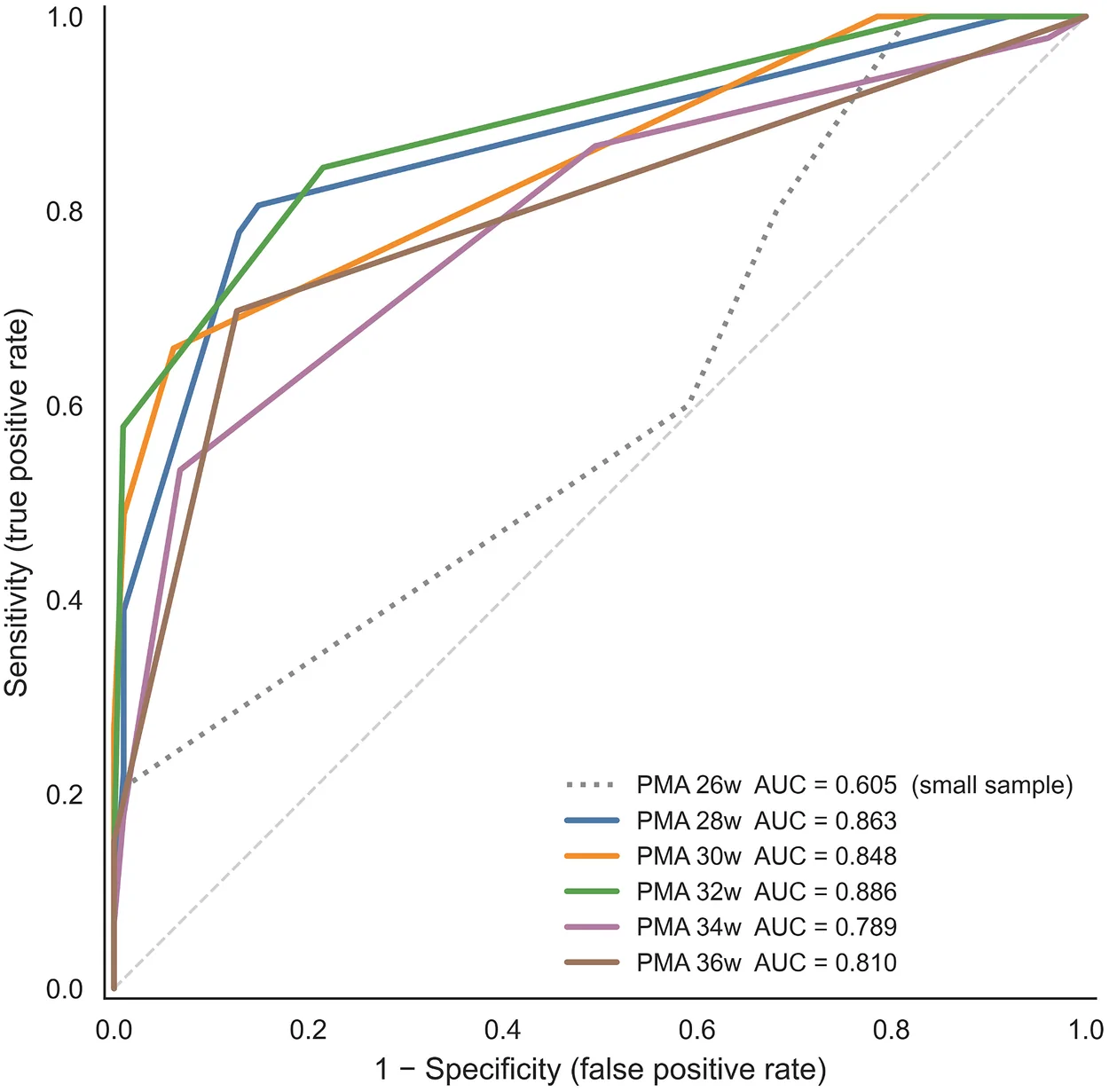
Apparent within-cohort discrimination between imaging-defined groups by Burdjalov total score at each PMA window. Receiver-operating-characteristic curves were generated separately within each pre-specified PMA window using the negative Burdjalov total score as the discriminator because lower scores were associated with membership in the brain-injury group. The curve at PMA 26 weeks is shown in grey because only 5 of the 27 infants contributing a recording were in the brain-injury group and should be interpreted with caution. Areas under the curve and 95% bootstrap confidence intervals are reported in Supplementary Table S9. AUC, area under the receiver-operating-characteristic curve; PMA, postmenstrual age.

Receiver-operating-characteristic analyses quantified apparent retrospective within-cohort discrimination between the two imaging-defined groups at each PMA window. The negative total score was used as the discriminator because lower scores were associated with the brain-injury group. For 5000 joint bootstrap draws (25), infants were resampled with replacement within imaging group and the same patient-level draw was applied across all PMA windows, preserving within-infant and cross-window dependence. Percentile confidence intervals were calculated for each PMA-specific area under the curve (AUC) and for paired differences between PMA 32 weeks and every other window (Supplementary Table S9 and Figure 4). Youden-derived cut-offs, sensitivity, and specificity were retained only as descriptive in-sample summaries and were not regarded as validated predictive, diagnostic, screening, or clinical-decision thresholds. The reference group's median subscore profile was tabulated as a descriptive maturation profile (Supplementary Figure S4).

#### 2.7.7 Sensitivity analyses

Variable-level missingness is summarized in Supplementary Figure S1. Continuous-scale analyses included complete-case restriction; restriction to infants contributing at least four PMA windows; categorical-PMA modeling; adjustment for gestational age, birth weight, and sex; Gaussian GEE; and two SGA-adjusted mixed models using the WS/T 800–2022 and Fenton 2025 classifications (Supplementary Table S5). In SGA-adjusted models, birth weight was omitted to avoid collinearity because SGA is derived from birth weight, sex, and gestational age. Reviewer-requested robustness analyses additionally included the cumulative ordinal GEE, the complete interaction-window sensitivity analysis, ceiling quantification, residual diagnostics, and the joint patient-level AUC bootstrap.

#### 2.7.8 Multiplicity and reporting

*P*-values are two-sided and reported to three decimal places, with values below 0.001 displayed as <0.001. No formal adjustment for multiplicity was applied across the four subscores or the six PMA windows; subscore and per-PMA analyses are interpreted as descriptive and hypothesis-generating, with the LMM PMA×group interaction on total score serving as the primary hypothesis test.

Reliability and inflammatory-exposure analyses. Exact agreement, agreement within one point, quadratic-weighted Cohen's *κ*, and absolute-agreement single-measure ICC(A,1) were calculated for the total score and each subscore. Confidence intervals used 2000 infant-level cluster-bootstrap resamples. Postnatal systemic sepsis and maternal infection/chorioamnionitis were compared descriptively between imaging groups using Fisher's exact test and were not entered into the primary longitudinal model.

### 2.8 Software and data availability

All analyses were performed in Python 3.12.3 using statsmodels 0.14.5 for the linear mixed-effects model and the generalized estimating equations (26), scikit-learn 1.7.2 for the receiver-operating characteristic analysis (27), pandas 2.3.3, and numpy 1.26.4. The analysis code is publicly available at https://github.com/MdXuDeKai/AEEG. De-identified aggregate analytic outputs are available from the corresponding author upon reasonable request; individual-level data are not publicly available because of institutional privacy restrictions and Chinese health-data regulations.

## 3 Results

### 3.1 Cohort characteristics and aEEG data availability

Of 274 screened infants, 98 did not contribute analyzable longitudinal data: life-sustaining treatment was withdrawn at the family's request in 49, 8 died despite full intensive care, and 41 did not accumulate three qualifying recordings. In 14 of these 41 infants, data collection closed before a third PMA window was reached; in the remaining 27, the reason could not be reliably reconstructed. The remaining 176 infants underwent formal eligibility assessment. After exclusion of 20 infants (necrotizing enterocolitis during the monitoring period, *n* = 2; intracranial infection during the monitoring period, *n* = 2; clinical or electrographic seizures, *n* = 1; incomplete clinical or imaging data, *n* = 15), 156 infants formed the final analytic cohort (Figure 1 and Supplementary Table S19). The cohort comprised 109 reference/no major brain injury infants and 47 brain-injury infants, including 21 with grade III–IV intraventricular hemorrhage and 26 with white-matter injury. After removal of 8 exact-duplicate records from 731 eligible longitudinal recording entries, the analytic dataset comprised 723 recordings: 27 at PMA 26 weeks, 137 at 28 weeks, 139 at 30 weeks, 152 at 32 weeks, 148 at 34 weeks, and 120 at 36 weeks (Supplementary Table S2).

Baseline perinatal characteristics did not differ significantly between the brain-injury and reference groups (all *p* > 0.05; Table 1), including gestational age, birth weight, sex, antenatal corticosteroid exposure, mode of delivery, assisted reproduction, and the small-for-gestational-age proportion by the WS/T 800–2022 classification (*p* = 0.296).

Within the reference group, 9/109 infants (8.3%) had normal imaging, 3/109 (2.8%) had grade I intraventricular hemorrhage, and 97/109 (89.0%) had grade II intraventricular hemorrhage (Supplementary Table S18). Postnatal systemic sepsis was recorded in 43/109 reference infants (39.4%) and 19/47 brain-injury infants (40.4%); maternal infection/chorioamnionitis was recorded in 6/109 (5.5%) and 3/47 (6.4%), respectively (both Fisher exact *p* = 1.000).

The reliability sample comprised 219 recordings from 45 infants. For the total score, inter-rater exact agreement was 91.8%, agreement within one point was 98.6%, weighted *κ* was 0.994 (95% cluster-bootstrap CI 0.990 to 0.997), and ICC(A,1) was 0.994. Inter-rater reliability results for all subscores are reported in Supplementary Table S17.

### 3.2 Descriptive maturation trajectories of the Burdjalov total score

In the reference group, the Burdjalov total score increased steadily with advancing PMA, with group-specific medians of 2 [interquartile range (IQR) 2–4] at PMA 26 weeks, 6 (IQR 6–6) at 28 weeks, 8 (IQR 8–8) at 30 weeks, 10 (IQR 10–10) at 32 weeks, 12 (IQR 11–12) at 34 weeks, and 13 (IQR 13–13) at 36 weeks (Supplementary Table S7 and Figure 2A; individual infant trajectories are shown in Supplementary Figure S2). The reference group approached ceiling values by PMA 34–36 weeks, where the within-group interquartile range collapsed to a single integer.

The brain-injury group followed a similarly increasing but consistently lower trajectory, with medians of 2 (IQR 2–3) at PMA 26 weeks, 4 (IQR 3–4) at 28 weeks, 7 (IQR 5–8) at 30 weeks, 8 (IQR 8–9) at 32 weeks, 10 (IQR 10–11) at 34 weeks, and 12 (IQR 12–13) at 36 weeks. Between-group differences in the total score were not statistically significant at PMA 26 weeks (*p* = 0.449), a finding to be interpreted with caution given the limited sample at this earliest window (*n* = 27; brain injury *n* = 5), but reached significance at every subsequent window from PMA 28 to 36 weeks (all *p* < 0.001 by the Mann–Whitney *U*-test; Supplementary Table S7). The reference-group values were subsequently tabulated as a preliminary descriptive maturation profile (section 3.8).

### 3.3 Domain-specific subscore trajectories

The four Burdjalov subscores displayed distinct between-group patterns across PMA (Figure 2B–E and Supplementary Table S1), indicating domain-specific maturational delay rather than a uniform downward shift in the brain-injury group.

Sleep–wake cycling showed the most persistent between-group difference, with significant differences at every PMA window from 28 to 36 weeks (all *p* < 0.001). The reference group advanced in a stepwise pattern across successive two-week PMA windows, from a median of 0 at PMA 26 weeks to 5 at 36 weeks, whereas the brain-injury group trailed by approximately one integer step at each subsequent window.

Bandwidth differences were present from PMA 28 to 36 weeks, although separation was small at the final window (*p* = 0.029 at PMA 36). Lower-border differences were significant from PMA 28 through 32 weeks and attenuated thereafter as both groups approached the maximum score of 2. Continuity differences were significant at PMA 28 and 30 weeks; from PMA 32 weeks onward, both groups had reached the maximum value of 2. The complete marginal distributions clarified comparisons in which medians and interquartile ranges were identical: at PMA 28, continuity scores 0/1/2 occurred in 1/90/10 reference recordings and 6/30/0 brain-injury recordings; at PMA 36, bandwidth scores 3/4 occurred in 2/85 and 4/29 recordings, respectively (Supplementary Table S15). In the exploratory subscore model, the sleep–wake cycling interaction was negative (−0.025 points/week, *p* = 0.038, unadjusted across four subscores), indicating slight widening rather than narrowing of the injury-minus-reference difference (Supplementary Table S4).

### 3.4 Primary longitudinal mixed-effects model

The primary linear mixed-effects model estimated a 0.92-point weekly increase in total score (95% CI 0.89 to 0.94, *p* < 0.001) and a −1.45-point brain-injury-group difference at PMA 32 weeks (95% CI −1.65 to −1.25, *p* < 0.001; Supplementary Table S8). The continuous-scale interaction was +0.111 points/week (95% CI 0.058 to 0.164, *p* < 0.001). However, the interaction was not robust to outcome scale or analysis window: the cumulative ordinal GEE interaction was −0.002 (*p* = 0.979), while the continuous-scale interaction ranged from +0.033 (*p* = 0.538) for PMA 26–32 to +0.128 (*p* = 0.023) for PMA 28–32 and +0.189 (*p* = 0.078) for PMA 30–32 (Supplementary Table S13). The interaction was therefore treated as specification-sensitive and was not interpreted as biological catch-up maturation.

The estimated group difference at PMA 32 weeks remained stable across the continuous-scale sensitivity analyses, ranging from −1.43 to −1.48 points; the categorical-PMA estimate at the same window was −1.54 points (Supplementary Table S5). Thus, the association between major brain injury and lower score was robust, whereas the PMA×group interaction was model- and window-dependent. Exact ceiling was absent through PMA 32 but accumulated at later windows; at PMA 36, 76/87 reference recordings (87.4%) and 9/33 brain-injury recordings (27.3%) reached the maximum score of 13 (Supplementary Table S14 and Figure S7).

Residual dispersion differed across PMA windows (descriptive Brown–Forsythe *p* < 0.001), supporting the use of PMA-stratified diagnostics and the ordinal sensitivity analysis. The random-slope model converged but was near-singular. Random-intercept variance ratios were approximately 0.000 in the intercept-only model, 0.420 after accounting for PMA, 0.151 after additionally accounting for imaging group, and 0.148 in the full interaction model (Supplementary Table S8), demonstrating that this variance partition is conditional on the fixed-effect specification.

### 3.5 PMA-specific group contrasts

To examine how the between-group difference evolved across the maturation window, linear contrasts of the categorical-PMA interaction model were used to derive a group-effect estimate at each PMA window (Figure 3; numerical values in Supplementary Table S6). The estimated difference was not significant at PMA 26 weeks (−0.64 points, 95% CI −1.51 to +0.24, *p* = 0.154), consistent with the small sample at that window and the wide confidence interval crossing the null.

Across the full observation period, the categorical-PMA group difference was non-monotonic: the imprecise PMA 26 estimate was −0.64 points (95% CI −1.51 to +0.24), followed by the largest observed separation at PMA 28 (−2.06, 95% CI −2.41 to −1.71). The difference then became progressively smaller at PMA 30 (−1.93), 32 (−1.54), 34 (−1.15), and 36 weeks (−0.91), with all confidence intervals from PMA 28 onward excluding zero (Supplementary Table S6). A single linear interaction therefore did not fully represent the observed shape.

### 3.6 Apparent within-cohort discrimination between imaging-defined groups across PMA windows

Receiver-operating-characteristic analyses quantified apparent retrospective within-cohort discrimination between the imaging-defined groups (Supplementary Table S9 and Figure 4). The largest observed AUC was at PMA 32 weeks (0.886, 95% bootstrap CI 0.824 to 0.940), with estimates of 0.863 at PMA 28, 0.848 at PMA 30, 0.789 at PMA 34, and 0.810 at PMA 36 weeks. In the joint patient-level bootstrap, the PMA 32 AUC exceeded PMA 26 by 0.281 (95% CI 0.012 to 0.584) and PMA 34 by 0.097 (95% CI 0.014 to 0.189), but differences versus PMA 28, 30, and 36 weeks had confidence intervals crossing zero. These results did not establish an optimal monitoring window.

At PMA 26 weeks, only 5 of the 27 infants contributing a recording were in the brain-injury group (18.5%), compared with 47 of 156 infants (30.1%) in the overall cohort. The AUC estimate was 0.605 (95% CI 0.297 to 0.880). The small sample size, lower proportion of infants with brain injury, and wide confidence interval substantially limited the precision and interpretability of this earliest-window estimate.

Exploratory Youden-derived cut-offs and their in-sample sensitivity and specificity are provided in Supplementary Table S9. Because these values were optimized and evaluated in the same cohort, they should not be interpreted as validated predictive, diagnostic, screening, or clinical decision thresholds.

### 3.7 Exploratory injury-subtype comparison

In the exploratory subgroup analysis, infants with severe (grade III–IV) intraventricular hemorrhage (*n* = 21) and infants with white-matter injury (*n* = 26) both followed Burdjalov total-score trajectories below the reference group, with significant three-group differences at every PMA window from 28 to 36 weeks (Kruskal–Wallis *p* < 0.001). The two brain-injury subtypes did not differ significantly from each other at any window (Mann–Whitney U *p* > 0.05 at all timepoints; Supplementary Table S10 and Figure S5), and subscore comparisons showed no consistent between-subtype difference (Supplementary Table S11). Baseline characteristics were similar between the two subtypes (Supplementary Table S12). Both injury subtypes therefore showed comparably reduced trajectories, supporting the pre-specified pooled analysis.

### 3.8 Reference-group maturation profile

The reference group's median total and subscore profile across PMA is provided as a descriptive within-cohort maturation profile (Supplementary Figure S4). This profile recapitulated the total-score trajectory shown in Figure 2A and illustrated the earlier ceiling of lower-border amplitude and continuity relative to the more gradual maturation of sleep–wake cycling and bandwidth.

## 4 Discussion

### 4.1 Principal findings

In this single-center longitudinal cohort of 156 extremely preterm infants with 723 analyzable serial aEEG recordings, imaging-defined major brain injury was consistently associated with lower Burdjalov scores from PMA 28 to 36 weeks. The categorical-PMA difference was non-monotonic across the full period, with an imprecise estimate at PMA 26, the largest observed separation at PMA 28, and progressively smaller differences thereafter. Although the primary continuous-scale model yielded a positive PMA×group interaction, its magnitude and statistical significance varied across analysis windows and it was absent in the ordinal model. Late-window ceiling accumulation was substantial, especially in the reference group. Sleep–wake cycling remained lower in the brain-injury group and showed a small negative exploratory interaction, whereas continuity and lower-border scores reached their maxima earlier. Joint patient-level AUC comparisons did not establish PMA 32 as an optimal monitoring window.

### 4.2 Comparison with previous literature

The reference-group maturation trajectory described here is consistent with previously published Burdjalov score reference values in preterm cohorts (4–6). Burdjalov and colleagues reported a steady PMA-related increase in the composite score (4), and subsequent studies described broadly comparable maturation patterns (5, 6, 28, 29). In a previous report from our group, Luo et al. (18) described 488 aEEG recordings from 101 infants without major brain injury; these observations overlap with the present cohort. The current analysis adds 8 reference infants with 30 recordings and, critically, a distinct brain-injury cohort of 47 infants with 205 recordings. Its novel contribution is the longitudinal comparison of imaging-defined groups, including model-based group differences, specification-sensitive interaction analyses, and within-cohort discrimination, rather than another single-arm description of reference maturation.

Prior studies have linked abnormal early or term-equivalent aEEG features to brain injury and later neurodevelopmental outcomes in preterm infants (9, 10, 13, 14, 30). In extremely preterm infants specifically, lower Burdjalov scores during the first 72 h of life have been associated with cerebral injury and with less favorable neurobehavioral outcomes at term and at 5 to 7 years of age (10). The present study adds a longitudinal dimension by characterizing not the presence or absence of an abnormal pattern at a single timepoint, but the trajectory of functional maturation across six PMA windows, with the largest separation at PMA 28 weeks and progressive narrowing of the observed score difference thereafter. The relatively high apparent within-cohort discrimination estimate at PMA 32 weeks is consistent with studies showing that 32-week Burdjalov-scored aEEG correlates with term-equivalent MRI abnormalities (11) and may complement term MRI in forecasting 2-year neurodevelopmental outcomes (12). In the present cohort, relatively high apparent AUC estimates were observed across PMA 28–32 weeks, but these descriptive estimates do not establish an optimal monitoring window.

### 4.3 Mechanistic interpretation

The domain-specific patterns are biologically plausible, but the present associations cannot establish mechanism. Sleep–wake cycling is among the latest aEEG features to mature in preterm infants (4, 5) and was persistently lower in the brain-injury group. However, its exploratory PMA×group estimate was negative (−0.025 points/week), meaning the injury-minus-reference difference became slightly more negative with increasing PMA; this result does not support convergence and was not adjusted for testing four subscores. Continuity and lower-border amplitude reached their scale maxima earlier, mechanically limiting measurable late-window separation. Bandwidth remained different through PMA 36, but the final-window difference was small. These scale-specific patterns are compatible with delayed functional organization but do not identify a cellular, structural, or causal mechanism.

Perinatal and postnatal inflammation is a plausible, non-exclusive contributor to the functional immaturity observed here. In neonatal mice, repeated systemic IL-1*β* exposure disrupted oligodendrocyte maturation and produced persistent myelination deficits (31). In preterm fetal sheep, subclinical exposure to low-dose endotoxin blunted EEG maturation, with transient plasma IL-6 elevation and increased numbers of microglia and TNF-*α*-positive cells in periventricular white matter, without hemodynamic disturbance or overt cortical injury (32). In neonatal rats, LPS exposure on postnatal days 1–3 produced dysmature EEG features on postnatal days 7 and 14 despite unchanged cortical diffusion MRI measures (33). Pyramidal neuron arborization was already reduced at day 7, indicating microscopic dysmaturation (33). Human evidence is limited, observational, and heterogeneous (34–37). Preterm infants with systemic inflammation had lower four-component aEEG maturation scores at 35 weeks' PMA, and CSF IL-1*β* at symptom onset was inversely associated with the same-day score (34). White matter injury was also more frequent, so an association independent of structural brain injury cannot be inferred (34). Placental lesions reflecting a fetal inflammatory response were associated with absent sleep–wake cycling on postnatal day 1 (35). In infants born before 28 weeks monitored monthly from 28 to 36 weeks' PMA, sepsis was associated with acute burst suppression but not with a slower increase in maturation score (36). Inflammation may therefore affect maturation through processes not captured by lesion-based imaging (32, 33), but independence from microscopic or MRI-detectable injury remains unproven (33, 34). Depending on timing, inflammation may confound the association between imaging-defined injury and functional maturation or mediate effects of upstream perinatal or postnatal insults on maturation. Similar diagnosis-level infection proportions between our groups do not exclude differences in timing, severity, recurrence, or subclinical burden. Component-specific effects, particularly on bandwidth, remain unproven (34, 37).

Although severe intraventricular hemorrhage and white-matter injury were combined into a single brain-injury group, these injuries differ neurophysiologically: severe intraventricular hemorrhage involves focal hemorrhagic insult to the germinal matrix and subependymal zones, whereas white-matter injury reflects more diffuse damage to oligodendrocyte precursors and axonal integrity, and the two could in principle perturb cortical-network maturation and sleep–wake cycling through distinct pathways. In our exploratory subgroup analysis, however, the two subtypes followed comparable trajectories with no significant difference at any PMA window (Supplementary Tables S10–S12 and Figure S5). This may indicate convergence on a shared final common pathway of delayed functional maturation, or may simply reflect limited statistical power given the modest subgroup sizes; the comparability supports the pre-specified pooled analysis while leaving subtype-specific differences to be examined in larger, adequately powered cohorts.

The positive interaction from the primary continuous-scale model should not be interpreted as biological recovery or catch-up maturation. First, the observed group difference was non-monotonic across the full PMA range, with the smallest and most imprecise estimate at PMA 26, the largest separation at PMA 28, and narrowing only thereafter. Second, the interaction varied materially with the selected analysis window and was not reproduced under the ordinal specification. Third, 87.4% of reference-group recordings and 27.3% of brain-injury recordings reached the maximum total score at PMA 36, constraining measurable late-window separation. Together, these findings indicate that a single linear interaction is an unstable summary of a bounded, non-monotonic pattern and cannot distinguish biological change from scale compression or changes in concurrent clinical state.

### 4.4 Interpretation and research implications

The ROC findings should be interpreted as apparent retrospective within-cohort separation of imaging-defined groups rather than as evidence of prediction, diagnosis, screening, or clinical utility. These analyses also cannot determine whether lower aEEG scores were caused by structural injury: shared antecedents, including inflammatory exposure and overall illness severity, may contribute to both neuroimaging abnormalities and functional immaturity without a direct causal link between them. Although the largest observed AUC occurred at PMA 32 weeks and relatively high estimates were observed from PMA 28 to 32 weeks, these analyses do not establish an optimal monitoring window. The reference-group maturation profile is likewise descriptive and should not be considered a normative clinical standard. The present findings should therefore not be used to trigger neuroimaging, determine follow-up intensity, or guide decisions for individual infants. Prospective multicenter studies with prespecified monitoring schedules, independent external validation of the imaging-group discrimination findings, and longitudinal assessment of neurodevelopmental outcomes are required before clinical utility can be evaluated.

### 4.5 Strengths and limitations

The study has several strengths. It includes 723 analyzable serial recordings across six prespecified PMA windows in 156 infants born before 28 weeks' gestation. The primary group effect was examined using mixed models, categorical-PMA contrasts, an ordinal patient-clustered model, complete time-window sensitivity analyses, residual diagnostics, ceiling summaries, and a 5000-draw joint patient-level AUC bootstrap. The reliability substudy included independent blinded re-scoring by a second assessor, with infant-level cluster-bootstrap confidence intervals. Data preparation, quality control, adjudication, and analyses were implemented in a reproducible scripted pipeline and reported in accordance with STROBE.

Several limitations should be acknowledged. The single-center retrospective design limits generalizability and precluded prospective standardization of imaging surveillance. Although serial aEEG was part of routine NICU neuromonitoring rather than being initiated only for suspected seizures or acute neurological deterioration, recordings were obtainable only while infants remained hospitalized and monitoring was clinically feasible. Inter-rater reliability was high in the 219-recording independent re-scoring sample, but formal intra-rater repeatability was not assessed and this substudy did not include every recording. Brain-injury classification was abstracted from clinical radiology reports rather than independent blinded image re-review and used both cranial ultrasound and magnetic resonance imaging. Because the reference group predominantly comprised infants with isolated grade II IVH rather than normal neuroimaging, subtle functional alterations associated with low-grade IVH may have attenuated the observed differences between groups. The reference trajectory should therefore not be interpreted as a normative trajectory for infants with entirely normal neuroimaging. The PMA 26-week stratum was small and contained only 5 brain-injury recordings, limiting precision. The study did not assess trajectories from PMA 36 weeks to term-equivalent age. Future prospective studies should incorporate standardized term-equivalent inpatient or outpatient neurophysiological follow-up and measures with sufficient dynamic range at later PMA.

The analyzed cohort comprised 156 of 274 screened infants (56.9%), and the requirement for at least three eligible serial aEEG recordings creates selection and survivor bias. Infants in whom life-sustaining treatment was withdrawn and those who died before completing serial monitoring may have had greater illness severity and a higher burden of brain injury. Among the 41 infants who remained in the unit but did not accumulate three qualifying recordings, the reason could not be reliably reconstructed in 27. Infants in the brain-injury group also contributed fewer recordings than reference infants (mean 4.36 vs. 4.75, Mann–Whitney *p* = 0.010). The trajectories described here therefore apply primarily to infants who survived and remained sufficiently stable to undergo repeated monitoring and may under-represent the most fragile infants; this limits extrapolation to the broader extremely preterm population. Residual confounding by illness severity, respiratory and cardiovascular support, and concurrent intensive-care interventions also cannot be excluded.

Detailed data on concurrent NICU medications that can influence cortical background activity, including caffeine, vasoactive agents, and cyclooxygenase inhibitors used for patent ductus arteriosus, were not systematically retrieved, and residual pharmacological confounding cannot be excluded. Several considerations nonetheless argue against these exposures accounting for the observed group differences. Caffeine is administered to the large majority of very preterm infants (89% of those born below 32 weeks of gestation in a recent national cohort (38) and would therefore be expected to be broadly balanced across groups. Moreover, baseline perinatal characteristics did not differ between groups (Table 1), the group effect was consistent across all sensitivity analyses, and it followed a coherent maturational trajectory rather than the transient, state-dependent shifts characteristic of acute pharmacological effects.

Finally, the C3–C4 derivation may not capture regional or lateralized abnormalities outside this channel, and long-term neurodevelopmental outcomes were not available. Infection data were limited to diagnosis-level indicators: postnatal systemic sepsis occurred in 43/109 reference infants and 19/47 brain-injury infants, while maternal infection/chorioamnionitis occurred in 6/109 and 3/47, respectively. Complete infection timing relative to individual recordings and serial inflammatory-biomarker trajectories were unavailable, so unmeasured inflammatory burden and its potential roles as confounder or mediator remain. The Youden-derived cut-offs are descriptive in-sample estimates and are not validated clinical thresholds. Although two SGA-adjusted models reproduced the primary group effect, residual confounding by intrauterine growth restriction cannot be fully excluded.

## 5 Conclusion

Imaging-defined major brain injury was associated with persistently lower serial Burdjalov maturation scores. The positive continuous-scale PMA×group interaction was model- and window-dependent, was not reproduced by the ordinal analysis, and summarized a non-monotonic pattern on a scale with substantial late-window ceiling accumulation; it should not be interpreted as biological recovery. The ROC analyses describe retrospective within-cohort separation of imaging-defined groups and do not establish prediction, diagnosis, screening, optimal monitoring timing, or clinical utility. Prospective multicenter validation with standardized serial monitoring and longitudinal neurodevelopmental outcomes is required.

## Data Availability

The analysis code supporting the findings of this study is publicly available at https://github.com/MdXuDeKai/AEEG. De-identified aggregate analytic outputs are available from the corresponding authors upon reasonable request. Individual-level data are not publicly available because of institutional privacy restrictions and Chinese health-data regulations. Further inquiries can be directed to the corresponding authors.
