# Supplementary material for: Serial amplitude-integrated electroencephalography trajectories and brain-injury-associated functional immaturity in extremely preterm infants
Source: Front Pediatr. 2026 Aug 26;14:1912111. doi: 10.3389/fped.2026.1912111 (PMC13553765; doi:10.3389/fped.2026.1912111)
Supplement: Supplementary file 1 [file Supplementaryfile1.docx]

Supplementary Figure S1. Variable-level missingness.

**Legend.** Variable-level missingness in the analytic long-format aEEG dataset and patient-level baseline dataset. Long-format key variables were complete. The primary WS/T 800–2022 small-for-gestational-age variable was missing for one infant born at 23+6 weeks, outside the lower gestational-age limit of that standard. aEEG, amplitude-integrated electroencephalography; PMA, postmenstrual age; SGA, small for gestational age.

Supplementary Figure S2. Individual Burdjalov total-score trajectories.

**Legend.** Faint lines show individual infant trajectories of the Burdjalov total score across available PMA windows, displayed to illustrate within-group heterogeneity. Thick lines show group-specific median trajectories for the reference/no major brain injury and brain-injury groups. PMA, postmenstrual age.

Supplementary Figure S3. Distribution of available PMA windows per infant.

**Legend.** Distribution of the number of distinct PMA windows contributed per infant among the 156 infants in the final analytic cohort. PMA, postmenstrual age.

Supplementary Figure S4. Reference-group maturation profile.

**Legend.** Median Burdjalov total and subdomain scores across PMA 26–36 weeks in the reference/no major brain injury group. Cell colors indicate the relative score, calculated as the median score divided by the theoretical maximum for that row, whereas numbers inside cells show the raw median score. This profile is presented as a within-cohort descriptive summary of serial aEEG maturation in extremely preterm infants without major brain injury. It is not advanced as a normative reference standard or clinical decision tool without external validation. aEEG, amplitude-integrated electroencephalography; PMA, postmenstrual age.

Supplementary Figure S5. Exploratory injury-subtype comparison of Burdjalov maturation across postmenstrual age (PMA).

**Legend.** (A) Burdjalov total-score trajectories for the reference group (gray, solid line, filled circles), the severe (grade III–IV) intraventricular hemorrhage subgroup (red, dashed line, open squares), and the white-matter injury subgroup (blue, dotted line, open triangles). Symbols denote group medians at each PMA window and shaded bands denote the interquartile range; the number of recordings per group (reference / severe intraventricular hemorrhage / white-matter injury) at each window is shown below the x-axis. (B–E) Median trajectories of the four Burdjalov subscores—(B) continuity, (C) sleep–wake cycling, (D) lower-border amplitude, and (E) bandwidth—across PMA for the same three groups. The two brain-injury subtypes followed comparable trajectories with no statistically significant difference at any PMA window (all *p* > 0.05; Supplementary Tables S10 and S11), supporting the pre-specified pooled brain-injury analysis. Y-axes are fixed to the theoretical score ranges (total 0–13; continuity 0–2; sleep–wake cycling 0–5; lower border 0–2; bandwidth 0–4).

**Supplementary Figure S6. Residual diagnostics for the primary random-intercept linear mixed-effects model.**

Legend. Diagnostic plots for the primary random-intercept linear mixed-effects model of the Burdjalov total score, based on 723 aEEG recordings from 156 infants. (A) Standardized conditional residuals versus conditional fitted values for the full analytic sample, colored by imaging group. (B) Normal Q–Q plot of the standardized conditional residuals for the full analytic sample. (C) Standardized conditional residuals versus conditional fitted values stratified by the prespecified PMA window. (D) Normal Q–Q plots of standardized conditional residuals stratified by PMA window. Horizontal lines in panels A and C indicate zero. The discrete banding reflects the integer-valued, bounded nature of the 0–13 Burdjalov total score. Residual dispersion differed across PMA windows (Brown–Forsythe test, P < 0.001). aEEG, amplitude-integrated electroencephalography; PMA, postmenstrual age; Q–Q, quantile–quantile.

**Supplementary Figure S6 (continued).**

**Supplementary Figure S6 (continued).**

**Supplementary Figure S7. Ceiling accumulation in the Burdjalov total score by postmenstrual age and imaging group.**

Legend. Percentages of recordings at each prespecified PMA window with the exact maximum Burdjalov total score of 13 (solid lines with circles) or within one point of the maximum, defined as a total score ≥12 (dashed lines with crosses), shown separately for the reference/no major brain injury and brain-injury groups. The denominator was the number of available recordings within each PMA-by-group stratum. At PMA 36 weeks, 76 of 87 reference-group recordings (87.4%) and 9 of 33 brain-injury recordings (27.3%) had a total score of 13; 87 of 87 (100%) and 28 of 33 (84.8%), respectively, had a score ≥12. These distributions quantify substantial late-PMA ceiling accumulation in the bounded total score, particularly in the reference group. PMA, postmenstrual age.
