# Supplementary material for: Serial amplitude-integrated electroencephalography trajectories and brain-injury-associated functional immaturity in extremely preterm infants
Source: Front Pediatr. 2026 Aug 26;14:1912111. doi: 10.3389/fped.2026.1912111 (PMC13553765; doi:10.3389/fped.2026.1912111)
Supplement: Supplementary file 2 [file Supplementaryfile2.docx]

**Supplementary Table S1. Per-PMA Mann–Whitney p-values for the total score and four subscores.**

*Two-sided Mann–Whitney U tests compare the reference / no major brain injury group with the brain-injury group within each postmenstrual age (PMA) window.*

| **PMA (weeks)** | **Total score** | **Continuity** | **Sleep–wake cycling** | **Lower-border amplitude** | **Bandwidth** |
| --- | --- | --- | --- | --- | --- |
| 26 | 0.449 | 0.760 | 0.931 | 0.077 | 0.327 |
| 28 | <0.001 | <0.001 | <0.001 | <0.001 | <0.001 |
| 30 | <0.001 | <0.001 | <0.001 | <0.001 | <0.001 |
| 32 | <0.001 | 1.000 | <0.001 | <0.001 | <0.001 |
| 34 | <0.001 | 1.000 | <0.001 | 0.134 | <0.001 |
| 36 | <0.001 | 1.000 | <0.001 | 1.000 | 0.029 |

Reported P-values are unadjusted descriptive comparisons within each PMA window. They do not test the longitudinal PMA × group interaction. PMA, postmenstrual age.

**Supplementary Table S2. Distribution of distinct PMA timepoints contributed per infant.**

| **Distinct PMA windows per infant** | **Number of infants** | **Percentage** |
| --- | --- | --- |
| 3 | 13 | 8.3% |
| 4 | 48 | 30.8% |
| 5 | 78 | 50.0% |
| 6 | 17 | 10.9% |

Percentages are calculated among the 156 infants in the final analytic cohort. PMA, postmenstrual age.

**Supplementary Table S3. Burdjalov subscores by PMA window and group.**

*Values are group-specific medians (interquartile ranges). P-values are from two-sided Mann–Whitney U tests.*

| **Subscore** | **PMA (weeks)** | **Reference n** | **Reference median (IQR)** | **Brain-injury n** | **Brain-injury median (IQR)** | **P-value** |
| --- | --- | --- | --- | --- | --- | --- |
| Continuity | 26 | 22 | 0.0 (0.0, 1.0) | 5 | 0.0 (0.0, 1.0) | 0.760 |
| Continuity | 28 | 101 | 1.0 (1.0, 1.0) | 36 | 1.0 (1.0, 1.0) | <0.001 |
| Continuity | 30 | 98 | 2.0 (2.0, 2.0) | 41 | 2.0 (1.0, 2.0) | <0.001 |
| Continuity | 32 | 107 | 2.0 (2.0, 2.0) | 45 | 2.0 (2.0, 2.0) | 1.000 |
| Continuity | 34 | 103 | 2.0 (2.0, 2.0) | 45 | 2.0 (2.0, 2.0) | 1.000 |
| Continuity | 36 | 87 | 2.0 (2.0, 2.0) | 33 | 2.0 (2.0, 2.0) | 1.000 |
| Sleep–wake cycling | 26 | 22 | 0.0 (0.0, 0.0) | 5 | 0.0 (0.0, 0.0) | 0.931 |
| Sleep–wake cycling | 28 | 101 | 1.0 (1.0, 1.0) | 36 | 1.0 (0.0, 1.0) | <0.001 |
| Sleep–wake cycling | 30 | 98 | 2.0 (2.0, 2.0) | 41 | 1.0 (1.0, 2.0) | <0.001 |
| Sleep–wake cycling | 32 | 107 | 3.0 (3.0, 3.0) | 45 | 2.0 (2.0, 3.0) | <0.001 |
| Sleep–wake cycling | 34 | 103 | 4.0 (4.0, 4.0) | 45 | 3.0 (3.0, 4.0) | <0.001 |
| Sleep–wake cycling | 36 | 87 | 5.0 (5.0, 5.0) | 33 | 4.0 (4.0, 5.0) | <0.001 |
| Lower border | 26 | 22 | 1.0 (1.0, 1.0) | 5 | 1.0 (1.0, 1.0) | 0.077 |
| Lower border | 28 | 101 | 2.0 (2.0, 2.0) | 36 | 1.0 (1.0, 1.2) | <0.001 |
| Lower border | 30 | 98 | 2.0 (2.0, 2.0) | 41 | 2.0 (1.0, 2.0) | <0.001 |
| Lower border | 32 | 107 | 2.0 (2.0, 2.0) | 45 | 2.0 (2.0, 2.0) | <0.001 |
| Lower border | 34 | 103 | 2.0 (2.0, 2.0) | 45 | 2.0 (2.0, 2.0) | 0.134 |
| Lower border | 36 | 87 | 2.0 (2.0, 2.0) | 33 | 2.0 (2.0, 2.0) | 1.000 |
| Bandwidth | 26 | 22 | 1.0 (1.0, 1.8) | 5 | 1.0 (1.0, 1.0) | 0.327 |
| Bandwidth | 28 | 101 | 2.0 (2.0, 2.0) | 36 | 1.0 (1.0, 1.2) | <0.001 |
| Bandwidth | 30 | 98 | 2.0 (2.0, 2.0) | 41 | 2.0 (1.0, 2.0) | <0.001 |
| Bandwidth | 32 | 107 | 3.0 (3.0, 3.0) | 45 | 2.0 (2.0, 2.0) | <0.001 |
| Bandwidth | 34 | 103 | 4.0 (3.0, 4.0) | 45 | 3.0 (3.0, 3.0) | <0.001 |
| Bandwidth | 36 | 87 | 4.0 (4.0, 4.0) | 33 | 4.0 (4.0, 4.0) | 0.029 |

Subscores are bounded as follows: continuity 0–2, sleep–wake cycling 0–5, lower-border amplitude 0–2, bandwidth 0–4. Non-integer interquartile-range bounds reflect linear interpolation across discrete-score percentiles. PMA, postmenstrual age.

**Supplementary Table S4. Linear mixed-effects models for the four Burdjalov subscores.**

*Parallel random-intercept linear mixed-effects models were fitted for each subscore. PMA was centered at 32 weeks; group was coded reference = 0 and brain injury = 1.*

| **Subscore** | **Fixed effect** | **Estimate** | **SE** | **95% CI** | **P-value** |
| --- | --- | --- | --- | --- | --- |
| Continuity | Intercept (PMA 32 weeks, reference group) | 1.781 | 0.016 | 1.750 to 1.813 | <0.001 |
| Continuity | PMA, per week | 0.122 | 0.005 | 0.112 to 0.133 | <0.001 |
| Continuity | Brain-injury group (vs reference at PMA 32 weeks) | −0.070 | 0.030 | −0.129 to −0.011 | 0.020 |
| Continuity | PMA × group, per week | 0.022 | 0.010 | 0.001 to 0.042 | 0.040 |
| Sleep–wake cycling | Intercept (PMA 32 weeks, reference group) | 2.970 | 0.026 | 2.920 to 3.020 | <0.001 |
| Sleep–wake cycling | PMA, per week | 0.470 | 0.006 | 0.458 to 0.483 | <0.001 |
| Sleep–wake cycling | Brain-injury group (vs reference at PMA 32 weeks) | −0.558 | 0.047 | −0.651 to −0.466 | <0.001 |
| Sleep–wake cycling | PMA × group, per week | −0.025 | 0.012 | −0.049 to −0.001 | 0.038 |
| Lower border | Intercept (PMA 32 weeks, reference group) | 1.951 | 0.016 | 1.919 to 1.983 | <0.001 |
| Lower border | PMA, per week | 0.032 | 0.004 | 0.023 to 0.041 | <0.001 |
| Lower border | Brain-injury group (vs reference at PMA 32 weeks) | −0.240 | 0.030 | −0.299 to −0.182 | <0.001 |
| Lower border | PMA × group, per week | 0.083 | 0.009 | 0.066 to 0.100 | <0.001 |
| Bandwidth | Intercept (PMA 32 weeks, reference group) | 2.919 | 0.022 | 2.875 to 2.963 | <0.001 |
| Bandwidth | PMA, per week | 0.291 | 0.007 | 0.277 to 0.305 | <0.001 |
| Bandwidth | Brain-injury group (vs reference at PMA 32 weeks) | −0.577 | 0.042 | −0.659 to −0.495 | <0.001 |
| Bandwidth | PMA × group, per week | 0.035 | 0.014 | 0.008 to 0.062 | 0.012 |

Models were fitted by restricted maximum likelihood with a patient-specific random intercept, PMA centered at 32 weeks, and group coded reference = 0 and brain injury = 1. For sleep–wake cycling, the negative interaction estimate (−0.025 points/week) indicates that the injury-minus-reference difference became more negative with increasing PMA; it does not indicate convergence. The estimated slopes were 0.470 points/week in the reference group and 0.445 points/week in the brain-injury group. Subscore analyses were exploratory, and interaction P-values were not adjusted across the four subscores. CI, confidence interval; PMA, postmenstrual age; SE, standard error.

**Supplementary Table S5. Sensitivity analyses for the primary group effect at PMA 32 weeks.**

These specifications primarily assess the robustness of the PMA and imaging-group effects on the continuous/Gaussian score scale. The bounded ordinal GEE and both early-window analyses are reported separately in Supplementary Table S13; together, the analyses support stable PMA and group effects but model- and window-dependent evidence for the PMA × group interaction.

| **Analysis** | **Infants** | **Records** | **PMA slope (95% CI)** | **Group effect at PMA 32 (95% CI)** | **PMA × group interaction (95% CI)** | **P (group)** | **P (interaction)** | **Note** |
| --- | --- | --- | --- | --- | --- | --- | --- | --- |
| Complete case (records with non-missing outcome) | 156 | 723 | 0.915 (0.888 to 0.942) | −1.448 (−1.646 to −1.249) | 0.111 (0.058 to 0.164) | <0.001 | <0.001 | Complete-case analytic frame. |
| ≥4 PMA timepoints per infant | 143 | 684 | 0.914 (0.888 to 0.941) | −1.477 (−1.685 to −1.268) | 0.093 (0.039 to 0.146) | <0.001 | <0.001 | Infants with ≥4 distinct PMA timepoints only. |
| PMA as categorical | 156 | 723 | — | −1.542 (−1.867 to −1.216) | — | <0.001 | — | Does not assume linear PMA effect; group effect at each PMA level is reported in Supplementary Table S6. |
| Adjusted (GA + birth weight + sex) | 156 | 723 | 0.915 (0.888 to 0.942) | −1.454 (−1.651 to −1.257) | 0.110 (0.057 to 0.163) | <0.001 | <0.001 | Adjusted for GA, birth weight and sex. |
| GEE (exchangeable, robust SE) | 156 | 723 | 0.915 (0.888 to 0.943) | −1.447 (−1.698 to −1.196) | 0.112 (0.040 to 0.184) | <0.001 | 0.002 | Population-averaged estimator with cluster-robust SE. |
| SGA-adjusted (WS/T 800–2022) | 155 | 717 | 0.912 (0.885 to 0.939) | −1.428 (−1.629 to −1.227) | 0.113 (0.060 to 0.165) | <0.001 | <0.001 | Adjusted for GA, sex and SGA defined by WS/T 800–2022; one infant at 23+6 weeks was unclassified and excluded from this model only. |
| SGA-adjusted (Fenton 2025) | 156 | 723 | 0.914 (0.887 to 0.941) | −1.445 (−1.644 to −1.246) | 0.110 (0.058 to 0.163) | <0.001 | <0.001 | Adjusted for GA, sex and SGA defined by Fenton 2025 size-at-birth cutoffs. |

In the WS/T 800–2022 row, one infant born at 23+6 weeks was outside the reference standard's gestational-age range and was excluded from that model only. GA, gestational age; GEE, generalized estimating equations; SE, standard error; SGA, small for gestational age.

**Supplementary Table S6. Per-PMA group-effect estimates from the categorical-PMA mixed-effects model.**

*Numerical companion to the per-PMA forest plot (main Figure 3). Each estimate represents brain-injury group minus reference group at the indicated PMA window.*

| **PMA (weeks)** | **Estimate** | **SE** | **95% CI** | **P-value** |
| --- | --- | --- | --- | --- |
| 26 | −0.638 | 0.447 | −1.514 to 0.239 | 0.154 |
| 28 | −2.060 | 0.181 | −2.414 to −1.706 | <0.001 |
| 30 | −1.927 | 0.174 | −2.267 to −1.587 | <0.001 |
| 32 | −1.542 | 0.166 | −1.867 to −1.216 | <0.001 |
| 34 | −1.149 | 0.167 | −1.476 to −0.821 | <0.001 |
| 36 | −0.910 | 0.190 | −1.283 to −0.538 | <0.001 |

Negative estimates indicate lower scores in the brain-injury group. CI, confidence interval; PMA, postmenstrual age; SE, standard error.

**Supplementary Table S7. Burdjalov total score by PMA window and imaging group.**

*Values are group-specific medians (interquartile ranges). P-values are from two-sided Mann–Whitney U tests.*

| **PMA (weeks)** | **Reference n** | **Reference median (IQR)** | **Brain-injury n** | **Brain-injury median (IQR)** | **P-value** |
| --- | --- | --- | --- | --- | --- |
| 26 | 22 | 2.0 (2.0, 4.0) | 5 | 2.0 (2.0, 3.0) | 0.449 |
| 28 | 101 | 6.0 (6.0, 6.0) | 36 | 4.0 (3.0, 4.0) | <0.001 |
| 30 | 98 | 8.0 (8.0, 8.0) | 41 | 7.0 (5.0, 8.0) | <0.001 |
| 32 | 107 | 10.0 (10.0, 10.0) | 45 | 8.0 (8.0, 9.0) | <0.001 |
| 34 | 103 | 12.0 (11.0, 12.0) | 45 | 10.0 (10.0, 11.0) | <0.001 |
| 36 | 87 | 13.0 (13.0, 13.0) | 33 | 12.0 (12.0, 13.0) | <0.001 |

Values are medians (interquartile ranges) of recordings within each PMA window and imaging group. P-values are from two-sided Mann–Whitney U tests and are descriptive cross-sectional comparisons. The analytic dataset comprised 723 recordings from 156 infants, with at most one recording per infant in each PMA window. PMA, postmenstrual age.

**Supplementary Table S8. Primary random-intercept model and random-effects diagnostics for the Burdjalov total score.**

*Random-intercept linear mixed-effects model fitted by restricted maximum likelihood. PMA was centered at 32 weeks; group was coded reference = 0 and brain injury = 1.*

| **Fixed effect** | **Estimate** | **SE** | **95% CI** | **P-value** |
| --- | --- | --- | --- | --- |
| Intercept (PMA 32 weeks, reference group) | 9.624 | 0.055 | 9.516 to 9.731 | <0.001 |
| PMA, per week | 0.915 | 0.014 | 0.888 to 0.942 | <0.001 |
| Brain-injury group (vs reference at PMA 32 weeks) | −1.448 | 0.101 | −1.646 to −1.249 | <0.001 |
| PMA × group, per week | 0.111 | 0.027 | 0.058 to 0.164 | <0.001 |

**Panel B. Variance-ratio ladder across fixed-effect specifications**

| **Model specification** | **Between-infant variance** | **Within-infant variance** | **Variance ratio** | **Boundary note** |
| --- | --- | --- | --- | --- |
| Intercept only | 0.000 | 8.990 | 0.000 | Random-intercept variance approximately zero; boundary estimate. |
| PMA only | 0.613 | 0.849 | 0.420 | — |
| PMA + group | 0.152 | 0.854 | 0.151 | — |
| PMA + group + interaction | 0.146 | 0.836 | 0.148 | — |

**Panel C. Random-intercept-plus-slope diagnostic**

| **Model** | **Final convergence** | **Intercept variance** | **PMA-slope variance** | **Intercept–slope covariance** | **Correlation** | **Eigenvalue ratio** | **Near-singular** | **Role** |
| --- | --- | --- | --- | --- | --- | --- | --- | --- |
| Random intercept + PMA slope | Yes | 0.136 | 0.009 | −0.035 | −0.995 | 0.000573 | Yes | Diagnostic only |

The primary random-intercept model was fitted by restricted maximum likelihood with PMA centered at 32 weeks. The PMA coefficient of 0.915 points/week is the reference-group slope under the interaction model. The variance ratio changes from approximately 0.000 in the intercept-only model to 0.420 after accounting for PMA and to 0.148 in the full interaction model. This dependence on the fixed-effect specification is expected in a rapidly maturing longitudinal outcome and these ratios should not be interpreted as inter-rater or intra-rater reliability. The random-intercept-plus-slope model reached final convergence after the BFGS–L-BFGS–CG optimizer sequence, but its random-effects covariance was near-singular; it was retained only as a covariance-structure diagnostic and excluded from inference. CI, confidence interval; PMA, postmenstrual age; SE, standard error.

**Supplementary Table S9. Retrospective within-cohort discrimination of imaging-defined groups across PMA windows.**

*Retrospective within-cohort separation of the imaging-defined groups was summarized separately at each PMA window; lower Burdjalov scores corresponded to the brain-injury group.*

| **PMA (weeks)** | **Total n** | **Brain-injury n (%)** | **AUC (95% CI)** | **Youden-derived cut-off (≤, in-sample)** | **Sensitivity (in-sample)** | **Specificity (in-sample)** | **Valid bootstrap draws** |
| --- | --- | --- | --- | --- | --- | --- | --- |
| 26 | 27 | 5 (18.5%) | 0.605 (0.297–0.880) | ≤1 | 0.200 | 1.000 | 4978 |
| 28 | 137 | 36 (26.3%) | 0.863 (0.790–0.928) | ≤5 | 0.806 | 0.851 | 5000 |
| 30 | 139 | 41 (29.5%) | 0.848 (0.782–0.908) | ≤7 | 0.659 | 0.939 | 5000 |
| 32 | 152 | 45 (29.6%) | 0.886 (0.824–0.940) | ≤9 | 0.844 | 0.785 | 5000 |
| 34 | 148 | 45 (30.4%) | 0.789 (0.703–0.866) | ≤10 | 0.533 | 0.932 | 5000 |
| 36 | 120 | 33 (27.5%) | 0.810 (0.724–0.888) | ≤12 | 0.727 | 0.874 | 5000 |

**Panel B. Paired AUC differences: PMA 32 weeks minus each other window**

| **Comparison** | **Observed AUC difference** | **Paired-bootstrap 95% CI** | **Valid joint draws** |
| --- | --- | --- | --- |
| PMA 32 − PMA 26 | 0.281 | 0.012 to 0.584 | 4978 |
| PMA 32 − PMA 28 | 0.023 | −0.064 to 0.110 | 5000 |
| PMA 32 − PMA 30 | 0.038 | −0.030 to 0.105 | 5000 |
| PMA 32 − PMA 34 | 0.097 | 0.014 to 0.189 | 5000 |
| PMA 32 − PMA 36 | 0.076 | −0.021 to 0.173 | 5000 |

**Panel C. Numbers of infants shared between PMA windows**

| **PMA (weeks)** | **26** | **28** | **30** | **32** | **34** | **36** |
| --- | --- | --- | --- | --- | --- | --- |
| 26 | 27 | 25 | 24 | 27 | 25 | 20 |
| 28 | 25 | 137 | 124 | 133 | 129 | 107 |
| 30 | 24 | 124 | 139 | 136 | 133 | 104 |
| 32 | 27 | 133 | 136 | 152 | 144 | 117 |
| 34 | 25 | 129 | 133 | 144 | 148 | 114 |
| 36 | 20 | 107 | 104 | 117 | 114 | 120 |

Lower Burdjalov scores corresponded to the brain-injury group, and the negative total score was used as the ROC marker. In each of 5000 bootstrap draws, infants were resampled within imaging-defined group and the same patient-level draw was applied jointly to all PMA windows, preserving within-infant and cross-window dependence. PMA-specific percentile confidence intervals and PMA 32-minus-other-window differences were obtained from the same paired draws. At PMA 26, 4978 draws were valid because some resamples did not retain both imaging groups within that window. Youden-derived cut-offs, sensitivity, and specificity are supplementary in-sample descriptors and are not validated clinical thresholds. AUCs quantify retrospective within-cohort imaging-group separation and do not establish prediction, diagnosis, screening, or clinical utility. In Panel C, diagonal values are the numbers available at each window and off-diagonal values are the numbers contributing to both windows. AUC, area under the receiver-operating-characteristic curve; PMA, postmenstrual age.

**Supplementary Table S10. Burdjalov total score by postmenstrual age in the reference group and the two brain-injury subtypes.**

| **PMA (weeks)** | **Reference n** | **Reference median (IQR)** | **sIVH n** | **sIVH median (IQR)** | **WMI n** | **WMI median (IQR)** | **P (Kruskal–Wallis)** | **P (sIVH vs WMI)** |
| --- | --- | --- | --- | --- | --- | --- | --- | --- |
| 26 | 22 | 2 (2–4) | 3 | 2 (2–2) | 2 | 2 (2–3) | 0.727 | 1.000 |
| 28 | 101 | 6 (6–6) | 16 | 4 (2–6) | 20 | 4 (3–4) | <0.001 | 0.908 |
| 30 | 98 | 8 (8–8) | 19 | 8 (6–8) | 22 | 6 (5–7) | <0.001 | 0.085 |
| 32 | 107 | 10 (10–10) | 19 | 9 (8–9) | 26 | 8 (8–9) | <0.001 | 0.670 |
| 34 | 103 | 12 (11–12) | 21 | 11 (10–11) | 24 | 10 (10–11) | <0.001 | 0.085 |
| 36 | 87 | 13 (13–13) | 16 | 12 (12–13) | 17 | 12 (12–12) | <0.001 | 0.320 |

*IQR, interquartile range; PMA, postmenstrual age; sIVH, severe (grade III–IV) intraventricular hemorrhage; WMI, white-matter injury. The Kruskal–Wallis P-value compares the three groups; the sIVH-vs-WMI P-value is from the Mann–Whitney U test. P-values are unadjusted for multiple comparisons.*

**Supplementary Table S11. Subscore comparison between the severe intraventricular hemorrhage and white-matter injury subtypes by postmenstrual age.**

| **PMA (weeks)** | **Continuity** | **Sleep–wake cycling** | **Lower border** | **Bandwidth** |
| --- | --- | --- | --- | --- |
| 26 | 1.000 | 0.414 | 0.414 | 1.000 |
| 28 | 0.041 | 0.418 | 0.679 | 0.701 |
| 30 | 0.936 | 0.164 | 0.116 | 0.129 |
| 32 | 1.000 | 0.024 | 0.410 | 0.225 |
| 34 | 1.000 | 0.031 | 0.306 | 0.252 |
| 36 | 1.000 | 0.407 | 1.000 | 0.339 |

*Values are Mann–Whitney U P-values for the severe (grade III–IV) intraventricular hemorrhage versus white-matter injury contrast, unadjusted for multiple comparisons. No subscore differed between subtypes consistently across postmenstrual-age windows; the isolated nominal differences (continuity at 28 weeks; sleep–wake cycling at 32 and 34 weeks) were not significant after accounting for the number of comparisons.*

**Supplementary Table S12. Baseline characteristics of the two brain-injury subtypes.**

| **Characteristic** | **Severe IVH (n = 21)** | **White-matter injury (n = 26)** | **P-value** |
| --- | --- | --- | --- |
| Gestational age (weeks), median (IQR) | 26 (26–27) | 27 (26–27) | 0.114 |
| Birth weight (g), median (IQR) | 900 (830–1000) | 925 (758–1015) | 0.814 |
| Male sex, n (%) | 9 (42.9) | 11 (42.3) | 1.000 |
| Antenatal corticosteroids, n (%) | 9 (42.9) | 18 (69.2) | 0.084 |

*IVH, intraventricular hemorrhage; IQR, interquartile range. P-values are from the Mann–Whitney U test for continuous variables and the Fisher exact test for categorical variables.*

**Supplementary Table S13. Ordinal and early-window sensitivity analyses of the Burdjalov total score.**

**Panel A. Patient-clustered cumulative ordinal GEE**

| **Fixed effect** | **Log-odds estimate** | **Robust SE** | **Odds ratio (95% CI)** | **P-value** |
| --- | --- | --- | --- | --- |
| PMA, per week | 2.036 | 0.112 | 7.657 (6.152–9.530) | <0.001 |
| Brain-injury group (vs reference at PMA 32 weeks) | −3.111 | 0.251 | 0.045 (0.027–0.073) | <0.001 |
| PMA × group, per week | −0.002 | 0.069 | 0.998 (0.873–1.142) | 0.979 |

**Panel B. Random-intercept LMMs restricted to early PMA windows**

| **PMA window** | **Infants n** | **Records n** | **Fixed effect** | **Estimate** | **SE** | **95% CI** | **P-value** |
| --- | --- | --- | --- | --- | --- | --- | --- |
| PMA 26–32 weeks (≤32) | 156 | 455 | Intercept | 10.102 | 0.093 | 9.920 to 10.285 | <0.001 |
| PMA 26–32 weeks (≤32) | 156 | 455 | PMA, per week | 1.087 | 0.027 | 1.034 to 1.140 | <0.001 |
| PMA 26–32 weeks (≤32) | 156 | 455 | Brain-injury group | −1.704 | 0.172 | −2.042 to −1.366 | <0.001 |
| PMA 26–32 weeks (≤32) | 156 | 455 | PMA × group, per week | 0.033 | 0.054 | −0.072 to 0.139 | 0.538 |
| PMA 28–32 weeks | 156 | 428 | Intercept | 10.035 | 0.093 | 9.853 to 10.217 | <0.001 |
| PMA 28–32 weeks | 156 | 428 | PMA, per week | 1.036 | 0.030 | 0.978 to 1.094 | <0.001 |
| PMA 28–32 weeks | 156 | 428 | Brain-injury group | −1.582 | 0.171 | −1.917 to −1.248 | <0.001 |
| PMA 28–32 weeks | 156 | 428 | PMA × group, per week | 0.128 | 0.056 | 0.017 to 0.239 | 0.023 |

**Panel C. Complete interaction-window sensitivity analysis**

| **PMA window** | **Infants n** | **Records n** | **At exact ceiling** | **PMA × group** | **95% CI** | **P-value** |
| --- | --- | --- | --- | --- | --- | --- |
| PMA 26–36 (all) | 156 | 723 | 12.4% | 0.111 | 0.058 to 0.164 | <0.001 |
| PMA 26–34 | 156 | 603 | 0.8% | 0.093 | 0.021 to 0.164 | 0.011 |
| PMA 28–34 | 156 | 576 | 0.9% | 0.151 | 0.078 to 0.225 | <0.001 |
| PMA 26–32 | 156 | 455 | 0.0% | 0.033 | −0.072 to 0.139 | 0.538 |
| PMA 28–32 | 156 | 428 | 0.0% | 0.128 | 0.017 to 0.239 | 0.023 |
| PMA 30–32 | 155 | 291 | 0.0% | 0.189 | −0.021 to 0.399 | 0.078 |

The ordinal generalized estimating equation used a cumulative higher-category formulation, working-independence correlation, and patient-clustered robust sandwich standard errors. Odds ratios greater than 1 indicate greater odds of being in a higher total-score category. Thirteen threshold parameters were estimated but are omitted as nuisance cut-points. PMA was centered at 32 weeks. All prespecified and reviewer-relevant time windows are reported; none was selected according to statistical significance. The interaction varied materially with the selected window and was not reproduced under the ordinal specification. As shown in Supplementary Table S6, the categorical-PMA estimates were also non-monotonic: the imprecise PMA 26 estimate was followed by the largest observed group separation at PMA 28 and progressively smaller separations thereafter. A single linear interaction should therefore not be interpreted as biological catch-up maturation. GEE, generalized estimating equation; LMM, linear mixed-effects model; PMA, postmenstrual age; SE, standard error.

**Supplementary Table S14. Ceiling accumulation in the Burdjalov total score and subscores by PMA window and imaging group.**

**Panel A. Total-score ceiling and near-ceiling accumulation**

| **PMA (weeks)** | **Group** | **n** | **Total score = 13, n/N (%)** | **Total score ≥12, n/N (%)** | **Distance to ceiling, median (IQR)** |
| --- | --- | --- | --- | --- | --- |
| 26 | Reference | 22 | 0/22 (0.0%) | 0/22 (0.0%) | 11.0 (9.0–11.0) |
| 26 | Brain injury | 5 | 0/5 (0.0%) | 0/5 (0.0%) | 11.0 (10.0–11.0) |
| 28 | Reference | 101 | 0/101 (0.0%) | 0/101 (0.0%) | 7.0 (7.0–7.0) |
| 28 | Brain injury | 36 | 0/36 (0.0%) | 0/36 (0.0%) | 9.0 (9.0–10.0) |
| 30 | Reference | 98 | 0/98 (0.0%) | 0/98 (0.0%) | 5.0 (5.0–5.0) |
| 30 | Brain injury | 41 | 0/41 (0.0%) | 0/41 (0.0%) | 6.0 (5.0–8.0) |
| 32 | Reference | 107 | 0/107 (0.0%) | 2/107 (1.9%) | 3.0 (3.0–3.0) |
| 32 | Brain injury | 45 | 0/45 (0.0%) | 0/45 (0.0%) | 5.0 (4.0–5.0) |
| 34 | Reference | 103 | 4/103 (3.9%) | 52/103 (50.5%) | 1.0 (1.0–2.0) |
| 34 | Brain injury | 45 | 1/45 (2.2%) | 6/45 (13.3%) | 3.0 (2.0–3.0) |
| 36 | Reference | 87 | 76/87 (87.4%) | 87/87 (100.0%) | 0.0 (0.0–0.0) |
| 36 | Brain injury | 33 | 9/33 (27.3%) | 28/33 (84.8%) | 1.0 (0.0–1.0) |

**Panel B. Proportions at the theoretical maximum of each subscore**

| **Subscore (theoretical maximum)** | **PMA (weeks)** | **Reference at maximum, n/N (%)** | **Brain injury at maximum, n/N (%)** |
| --- | --- | --- | --- |
| Continuity (2) | 26 | 0/22 (0.0%) | 0/5 (0.0%) |
| Continuity (2) | 28 | 10/101 (9.9%) | 0/36 (0.0%) |
| Continuity (2) | 30 | 92/98 (93.9%) | 28/41 (68.3%) |
| Continuity (2) | 32 | 107/107 (100.0%) | 45/45 (100.0%) |
| Continuity (2) | 34 | 103/103 (100.0%) | 45/45 (100.0%) |
| Continuity (2) | 36 | 87/87 (100.0%) | 33/33 (100.0%) |
| Sleep-wake cycling (5) | 26 | 0/22 (0.0%) | 0/5 (0.0%) |
| Sleep-wake cycling (5) | 28 | 0/101 (0.0%) | 0/36 (0.0%) |
| Sleep-wake cycling (5) | 30 | 0/98 (0.0%) | 0/41 (0.0%) |
| Sleep-wake cycling (5) | 32 | 0/107 (0.0%) | 0/45 (0.0%) |
| Sleep-wake cycling (5) | 34 | 4/103 (3.9%) | 1/45 (2.2%) |
| Sleep-wake cycling (5) | 36 | 78/87 (89.7%) | 9/33 (27.3%) |
| Lower border (2) | 26 | 5/22 (22.7%) | 0/5 (0.0%) |
| Lower border (2) | 28 | 88/101 (87.1%) | 9/36 (25.0%) |
| Lower border (2) | 30 | 97/98 (99.0%) | 26/41 (63.4%) |
| Lower border (2) | 32 | 107/107 (100.0%) | 40/45 (88.9%) |
| Lower border (2) | 34 | 103/103 (100.0%) | 44/45 (97.8%) |
| Lower border (2) | 36 | 87/87 (100.0%) | 33/33 (100.0%) |
| Bandwidth (4) | 26 | 0/22 (0.0%) | 0/5 (0.0%) |
| Bandwidth (4) | 28 | 0/101 (0.0%) | 0/36 (0.0%) |
| Bandwidth (4) | 30 | 0/98 (0.0%) | 0/41 (0.0%) |
| Bandwidth (4) | 32 | 13/107 (12.1%) | 0/45 (0.0%) |
| Bandwidth (4) | 34 | 70/103 (68.0%) | 9/45 (20.0%) |
| Bandwidth (4) | 36 | 85/87 (97.7%) | 29/33 (87.9%) |

The Burdjalov total score ranges from 0 to 13. Exact ceiling was defined as a total score of 13, near ceiling as a total score of at least 12, and distance to ceiling as 13 minus the observed total score. Component ceilings used each subscore’s theoretical maximum. Percentages use the number of recordings within the corresponding PMA-by-group cell as the denominator. These summaries are descriptive and no independent-observation inference was performed. PMA, postmenstrual age.

**Supplementary Table S15. Full marginal score distributions for continuity at PMA 28 weeks and bandwidth at PMA 36 weeks.**

**Panel A. Full marginal distributions**

| **Outcome and PMA** | **Score** | **Reference n (%)** | **Brain-injury n (%)** |
| --- | --- | --- | --- |
| Continuity at PMA 28 weeks | 0 | 1 (1.0%) | 6 (16.7%) |
| Continuity at PMA 28 weeks | 1 | 90 (89.1%) | 30 (83.3%) |
| Continuity at PMA 28 weeks | 2 | 10 (9.9%) | 0 (0.0%) |
| Bandwidth at PMA 36 weeks | 0 | 0 (0.0%) | 0 (0.0%) |
| Bandwidth at PMA 36 weeks | 1 | 0 (0.0%) | 0 (0.0%) |
| Bandwidth at PMA 36 weeks | 2 | 0 (0.0%) | 0 (0.0%) |
| Bandwidth at PMA 36 weeks | 3 | 2 (2.3%) | 4 (12.1%) |
| Bandwidth at PMA 36 weeks | 4 | 85 (97.7%) | 29 (87.9%) |

**Panel B. Distribution-test summaries**

| **Outcome and PMA** | **Mann–Whitney U** | **Tie-corrected P-value** | **Rank-biserial correlation** |
| --- | --- | --- | --- |
| Continuity at PMA 28 weeks | 2253.0 | <0.001 | 0.239 |
| Bandwidth at PMA 36 weeks | 1576.5 | 0.029 | 0.098 |

Percentages are calculated within imaging group. P-values are from two-sided Mann–Whitney U tests using the normal approximation with tie correction. Positive rank-biserial correlations indicate a tendency toward higher scores in the reference group. Complete distributions are shown because medians and interquartile ranges can conceal differences when scores contain many ties. These comparisons are descriptive and were not adjusted for multiple testing. PMA, postmenstrual age.

**Supplementary Table S16. PMA-stratified conditional residual summaries and Brown–Forsythe diagnostics for the primary linear mixed-effects model.**

**Panel A. Conditional residual summaries**

| **PMA (weeks)** | **Group** | **n** | **Mean residual** | **SD** | **Median (IQR)** | **Median absolute residual** | **RMSE** |
| --- | --- | --- | --- | --- | --- | --- | --- |
| 26 | All | 27 | −0.790 | 1.338 | −0.940 (−1.940–0.102) | 1.804 | 1.532 |
| 26 | Reference | 22 | −1.042 | 1.333 | −1.817 (−1.940–−0.117) | 1.817 | 1.668 |
| 26 | Brain injury | 5 | 0.321 | 0.645 | 0.058 (0.048–0.547) | 0.367 | 0.660 |
| 28 | All | 137 | −0.200 | 0.954 | −0.045 (−0.288–0.142) | 0.229 | 0.971 |
| 28 | Reference | 101 | −0.157 | 0.792 | 0.003 (−0.152–0.142) | 0.142 | 0.803 |
| 28 | Brain injury | 36 | −0.320 | 1.312 | −0.314 (−1.067–0.291) | 0.898 | 1.333 |
| 30 | All | 139 | 0.288 | 0.947 | 0.231 (0.087–0.874) | 0.399 | 0.987 |
| 30 | Reference | 98 | 0.364 | 0.504 | 0.226 (0.125–0.448) | 0.311 | 0.619 |
| 30 | Brain injury | 41 | 0.108 | 1.559 | 0.652 (−0.646–1.442) | 1.333 | 1.543 |
| 32 | All | 152 | 0.298 | 0.674 | 0.342 (−0.032–0.574) | 0.453 | 0.735 |
| 32 | Reference | 107 | 0.327 | 0.581 | 0.359 (0.187–0.568) | 0.410 | 0.664 |
| 32 | Brain injury | 45 | 0.230 | 0.860 | 0.228 (−0.230–0.813) | 0.559 | 0.881 |
| 34 | All | 148 | 0.036 | 0.792 | 0.159 (−0.443–0.464) | 0.443 | 0.790 |
| 34 | Reference | 103 | 0.011 | 0.625 | 0.185 (−0.443–0.459) | 0.443 | 0.622 |
| 34 | Brain injury | 45 | 0.093 | 1.089 | 0.133 (−0.426–0.547) | 0.519 | 1.081 |
| 36 | All | 120 | −0.350 | 0.479 | −0.274 (−0.507–−0.099) | 0.327 | 0.591 |
| 36 | Reference | 87 | −0.379 | 0.341 | −0.274 (−0.460–−0.180) | 0.274 | 0.508 |
| 36 | Brain injury | 33 | −0.273 | 0.729 | −0.292 (−0.665–0.266) | 0.506 | 0.768 |

**Panel B. Brown–Forsythe diagnostics across PMA windows**

| **Scope** | **Test** | **Test statistic** | **P-value** |
| --- | --- | --- | --- |
| All | Brown-Forsythe (median-centered Levene) across PMA windows | 10.423 | <0.001 |
| Brain injury | Brown-Forsythe (median-centered Levene) across PMA windows | 3.393 | 0.006 |
| Reference | Brown-Forsythe (median-centered Levene) across PMA windows | 10.125 | <0.001 |

Conditional residuals were obtained from the primary random-intercept linear mixed-effects model. Brown–Forsythe tests are median-centered Levene diagnostics comparing residual dispersion across the six PMA windows. Because recordings were repeated within infants, these tests are presented as descriptive diagnostics rather than standalone inferential tests; PMA-stratified residual-versus-fitted and Q–Q plots remain the primary model checks. The marked variation in residual dispersion indicates that the Gaussian homoscedasticity assumption was not supported. PMA, postmenstrual age; Q–Q, quantile–quantile; RMSE, root mean square error; SD, standard deviation.

**Supplementary Table S17. Inter-rater reliability in the independent blinded re-scoring sample.**

**Panel A. Inter-rater score agreement**

| **Comparison** | **Score** | **Records n** | **Infants n** | **Exact agreement** | **Within 1 point** | **Weighted κ (95% CI)** | **ICC(A,1) (95% CI)** |
| --- | --- | --- | --- | --- | --- | --- | --- |
| Inter-rater | Continuity | 219 | 45 | 99.1% | 100.0% | 0.988 (0.970–1.000) | 0.988 (0.970–1.000) |
| Inter-rater | Sleep Cycle | 219 | 45 | 98.2% | 100.0% | 0.996 (0.992–0.999) | 0.996 (0.992–0.999) |
| Inter-rater | Lower Border | 219 | 45 | 98.2% | 100.0% | 0.939 (0.863–0.988) | 0.940 (0.863–0.988) |
| Inter-rater | Bandwidth | 219 | 45 | 95.4% | 99.5% | 0.973 (0.948–0.990) | 0.973 (0.948–0.990) |
| Inter-rater | Total | 219 | 45 | 91.8% | 98.6% | 0.994 (0.990–0.997) | 0.994 (0.990–0.997) |

**Panel B. Overall disagreement summary**

| **Comparison** | **Records / infants** | **Records with any disagreement** | **Mean total-score difference** | **SD of difference** | **Wilcoxon P-value** | **PMA windows** |
| --- | --- | --- | --- | --- | --- | --- |
| Inter-rater | 219 / 45 | 18 | 0.041 | 0.350 | 0.079 | 26,28,30,32,34,36 |

The 219-recording reliability sample included 45 infants across all six PMA windows. A second assessor independently re-scored these records while masked to imaging findings, imaging-group allocation, and the archived original scores. Inter-rater reliability compares the archived study scores with the second assessor's independent scores. Formal intra-rater repeatability was not assessed. Quadratic-weighted κ and absolute-agreement single-measure ICC(A,1) are reported. Confidence intervals were obtained from 2000 infant-level cluster-bootstrap resamples, preserving repeated recordings within infants. The original 723-recording dataset had been scored retrospectively in one centralized study-phase exercise; no calendar-time rater-drift analysis was applicable. CI, confidence interval; ICC, intraclass correlation coefficient; PMA, postmenstrual age; SD, standard deviation.

**Supplementary Table S18. Reference-group imaging composition and inflammatory-exposure descriptives.**

**Panel A. Reference-group imaging composition**

| **Imaging category** | **Infants n** | **Percentage** |
| --- | --- | --- |
| Normal imaging | 9 | 8.3% |
| Grade I IVH | 3 | 2.8% |
| Grade II IVH | 97 | 89.0% |

**Panel B. Inflammatory exposures by imaging group**

| **Exposure** | **Reference, n/N (%)** | **Brain injury, n/N (%)** | **Fisher exact P-value** | **Analysis role** |
| --- | --- | --- | --- | --- |
| Postnatal systemic sepsis diagnosis | 43/109 (39.4%) | 19/47 (40.4%) | 1.000 | Descriptive only; timing/biomarker trajectories incomplete and exposure may be a mediator. |
| Maternal infection / chorioamnionitis | 6/109 (5.5%) | 3/47 (6.4%) | 1.000 | Descriptive only; timing/biomarker trajectories incomplete and exposure may be a mediator. |

The reference group included infants with normal imaging or grade I–II intraventricular haemorrhage but no major brain injury. Systemic sepsis and maternal infection/chorioamnionitis are shown descriptively because complete timing relative to each aEEG recording and serial inflammatory-biomarker trajectories were not available, and postnatal infection may lie on the causal pathway between clinical illness and brain injury. These variables were therefore not added post hoc to the primary longitudinal model. Percentages use the number of infants in the corresponding imaging group as the denominator. aEEG, amplitude-integrated electroencephalography.

**Supplementary Table S19. Final study-flow accounting and overlap with the previously reported reference cohort.**

**Panel A. Study-flow accounting**

| **Stage** | **Infants n** | **Removed since prior stage** | **Reason** |
| --- | --- | --- | --- |
| Extremely preterm infants admitted/screened | 274 | 0 | Study-period admissions meeting the gestational-age scope. |
| At least three qualifying aEEGs; formal eligibility assessment | 176 | 98 | Did not contribute analyzable longitudinal data: life-sustaining treatment withdrawn at family request 49; died despite full intensive care 8; fewer than three qualifying recordings 41 (data collection closed before the third PMA window 14; reason not reliably reconstructable 27). |
| Final analytic cohort | 156 | 20 | Excluded: NEC 2; overlapping intracranial infection 2; seizure 1; incomplete clinical/imaging outcome ascertainment 15. |

**Panel B. Overlap with Reference 18**

| **Current cohort** | **Overlap from Ref. 18** | **Additional reference cohort** | **New brain-injury cohort** | **Total additional contribution** |
| --- | --- | --- | --- | --- |
| 156 infants / 723 recordings | 101 reference infants / 488 recordings; no brain-injury infant | 8 infants / 30 recordings | 47 infants / 205 recordings | 55 infants / 235 recordings |

Reference 18 reported 101 infants without major brain injury who contributed 488 aEEG recordings; these reference observations overlap with the present cohort. The present study includes 109 reference infants with 518 recordings and, critically, a distinct brain-injury cohort of 47 infants with 205 recordings. The present scientific question is the longitudinal comparison of maturation trajectories between imaging-defined groups, which was not addressed in Reference 18. Of 274 screened infants, 98 did not contribute analyzable longitudinal data; 176 entered formal eligibility assessment and 156 comprised the final analytic cohort after 20 eligibility exclusions.
